# Medicinal Plants Used Traditionally for Skin Related Problems in the South Balkan and East Mediterranean Region—A Review

**DOI:** 10.3389/fphar.2022.936047

**Published:** 2022-07-05

**Authors:** Efthymia Eleni Tsioutsiou, Vaios Amountzias, Argyro Vontzalidou, Evanthia Dina, Zora Dajić Stevanović, Antigoni Cheilari, Nektarios Aligiannis

**Affiliations:** ^1^ Department of Pharmacognosy and Natural Products Chemistry, Faculty of Pharmacy, National and Kapodistrian University of Athens, Athens, Greece; ^2^ Faculty of Agriculture, University of Belgrade, Belgrade, Serbia

**Keywords:** ethnopharmacology, skin, balkan peninsula, mediterranean, dioscorides, dermatological ailments, wound healing, anti-inflammatory

## Abstract

A review research was conducted to provide an overview of the ethnobotanical knowledge of medicinal plants and traditional medical practices for the treatment of skin disorders in Albania, Cyprus, Greece, and Turkey. The geographical and ecological characteristics of the Balkan Peninsula and Mediterranean Sea, along with the historical connection among those countries, gave rise to the development of a distinct flora and to the uses of common medicinal plants against various skin ailments, respectively. The review focuses on the detailed study of 128 ethnobotanical surveys conducted in these areas and the species used for skin ailments were singled out. The analysis showed that 967 taxa belonging to 418 different genera and 111 different families are used in the treatment of skin related problems. The majority of the plants belong to the families of Asteraceae (11.7%), Lamiaceae (7.4%), Rosaceae (6.7%), Plantaginaceae (5.4%), and Malvaceae (3.8%). Their usage is internal or external to treat ailments such as wounds and burns (22.1%), hemorrhoids (14.7%), boils, abscesses, and furuncles (8.2%). Beside specific skin disorders, numerous species appeared to be used for their antifungal, antimicrobial, and antiseptic activity (9.1%). Literature evaluation highlighted that, the most commonly used species are *Plantago major* L. (Albania, Turkey), *Hypericum perforatum* L. (Greece, Turkey), *Sambucus nigra* L. (Cyprus, Greece), *Ficus carica* L. (Cyprus, Turkey), *Matricaria chamomilla* L. (Cyprus, Greece), and *Urtica dioica* L. (Albania, Turkey), while many medicinal plants reported by interviewees were common in all four countries. Finally, to relate this ethnopharmacological knowledge and trace its expansion and diversification through centuries, a comparison of findings was made with the use of the species mentioned in Dioscorides’ “*De Materia Medica”* for skin disorders. This work constitutes the first comparative study performed with ethnobotanical data for skin ailments gathered in the South Balkan and East Mediterranean areas. Results confirm the primary hypothesis that people in Albania, Cyprus, Greece, and Turkey are closely related in terms of traditionally using folk medicinal practices. Nevertheless, more field studies conducted, especially in remote places of these regions, can help preserve the traditional medical knowledge, aiming at the discovery of new phytotherapeutics against dermatological diseases.

## Introduction

Herbal therapies have been used for the treatment of skin conditions for centuries in the Balkan countries, while several plant compounds are still used in topical treatments (Jarić et al., 2018). The most frequent categories for which medicinal plants and their preparations are used are wounds, hemorrhoids, boils, and eczema, while they are also commonly applied for their antibacterial and anti-inflammatory activity contributing to skin healing. For example, *Plantago major* L. is the most cited species for the treatment of traumas, wounds, and boils while *Urtica dioica* L. is principally mentioned to be applied topically against eczemas. Among the preparation forms, ointment, decoction, compress, and poultice are some of the most representative and regularly comprise the basis for the formulation of commercial products employed widely to cure skin ailments (e.g., Histoplastin Red^®^, Contractubex Gel^®^). The Balkan Peninsula and the Mediterranean Sea appertain to an area characterized by a high plant biodiversity and an important tradition in folk medicine. The diversity of the flora and the presence of endemism are strongly connected to the geographical position, the climate, and the geological composition (Varga et al., 2019; Emre et al., 2021). Phytogeographical analysis of the study area shows that 51% of the taxa are “narrows” (restricted to the Balkan Peninsula and Italy or the Balkan Peninsula and Anatolia), and 49% are more widely distributed (Strid, 1986). Environmental heterogeneity is high in the Mediterranean basin and this contributes to the high vascular plant species richness, especially in the eastern Mediterranean, due to evolutionary history and past climate. In particular, Last Glacial Maximum climate may have significantly shaped the current longitudinal and altitudinal patterns of species and genetic diversity trend in the Mediterranean (Fady and Conord, 2010). More specifically, all four countries included in the present review are divided in different phytogeographical regions. The floristic regions of Greece are 13 and are represented by North East, North Central, Northern Pindos, East Central, Southern Pindos, Ionian Islands, Sterea Ellas, West Aegean Islands, Peloponnisos, Kiklades, North Aegean Islands, East Aegean Islands, Kriti, and Karpathos (Annotations [Internet], 2022). Turkey has various macro/micro climates and vegetation types along with three overlapping phytogeographic regions represented by the Euro-Siberian, the Mediterranean, and the Irano-Turanian (Özşahin et al., 2019). This combination of geology and geography with topographic and climatic variation (Çolak and Rotherham, 2006) results in unusual levels of plant diversity and endemism. The phytogeographical divisions of Cyprus are 8 and are defined by the following regions: Akamas peninsula, Troodos range, the South area around Limassol, Larnaca area, the east part of Central plain, the west part of Central plain, the northern slopes and peaks of Pentadactylos, and Karpasia peninsula (Hadjichambis et al., 2004). The phytogeographical districts of Albania are represented by district of Berat, district of Burrel, district of Delvinë, district of Dibër, district of Elbasan, district of Kolonjë, district of Korçë, district of Lezhë, district of Librazhd, district of Mat, district of Përmet, district of Pogradec, district of Pukë, district of Sarandë, district of Tepelenë, district of Tiranë, district of Tropojë, and district of Vlorë (Barina and Pifkó, 2011). Despite the rich diversity and importance of flora as well as the presence of endemism in the study area, only a small proportion of the classified plants have been investigated and chemically characterized (Hoffmann et al., 2020). However, during the last years the therapeutic potential of an important number of medical plants traditionally used in dermatology has been explored, and some of them have been developed and approved as drugs or medical devices for the treatment of skin disorders (Tabassum and Hamdani, 2014). In defiance of all the prodigious advancements in modern phytochemical and medical research, ethnopharmacology of traditional medicinal plants in the Balkan and Southeast Mediterranean Region could be served as an important tool, providing a comprehensive approach to health systems in the countries of the area, preserving cultural diversity and strengthening the traditional medicine itself. The traditional practices and the ethnobotanical knowledge deriving from herbal manuscripts, could be exploited and used as a founding pillar, leading to the discovery of new bioactive natural products for the treatment of various problematic skin conditions. The aim of this review is to reveal, compare and contrast the traditional medical practices and the ethnobotanical knowledge of medicinal plants for the treatment of skin disorders in Albania, Cyprus, Greece, and Turkey. This was accomplished through a profound literature research on the ethnopharmacological field studies conducted in these four countries and through the listing of the information reported in order to collect the plant uses against problematic skin conditions. As a second target and to associate the bulk of ethnopharmacological data and confirm its expansion and diversification through centuries, we drew a parallel between the uses of the medicinal species reported against skin disorders in the articles we studied, and the ones mentioned in Dioscorides’ *De Materia Medica* for the same purpose.

### Skin Disorders

The skin represents the largest organ of the integumentary system with a surface of 2 m^2^. Its main function is to protect the underlying tissues such as muscles, bones, and internal organs. The skin is made up of a series of tissues of ectodermal and mesodermal origin and as a sequel of the orifices it continues with the respective mucous membranes forming a layer without interruptions. It is also characterized by an important distensibility and resistance (Anastasi et al., 2012). The skin acts as a protective envelope to the body and is closely connected to the underlying fascial endoskeleton through blood vessels, nerves, retinacular ligaments, and lymphatics. It consists of the epidermis which is mainly made of epithelial and is the most superficial and biologically active of the skin’s layers. As the basal layer of the epithelium (*stratum basale*) is constantly renewing. The second skin layer is the dermis which is considered to be the “core” of the integumentary system and provides most of the mechanical strength to the skin. The dermis is composed by the papillary and the reticular, both composed by connective tissue with fibers of collagen. Finally, the hypodermis, also called the subcutaneous layer, mainly consists of loose connective tissue and connects the skin to the underlying fibrous tissue of the bones and muscles (Wong et al., 2016). Skin disorders represent a very common problematic event and can affect all individuals during their life. Even a slight and superficial wound can lead to more serious pathological states, and trigger conditions that are difficult to control such as secondary bacterial infections, failure or abnormal progression of the healing process that promotes chronic wounds or scar formation both aesthetically and functionally altered. Since ancient times, all populations-including the Balkans-used various medicinal plants as a remedy against problematic skin ailments. Traditional medical practices have represented for hundreds of years the only resource for skin care, and still today maintain a very important role thanks to the multitasking characteristics possessed by the phytocomplex (Gertsch, 2011). Skin diseases are classified in various ways. One of these is based on the next three factors: 1) site of involvement such as facial rashes, lesions on sun-exposed sites, 2) pathogenesis such as genetic abnormalities, infectious etiology, or autoimmune mechanisms, 3) main structure affected such as epidermal diseases, abnormalities of melanocytes, and vascular changes. These good-standing categorizations are getting enriched as the science of dermatology expands and evolves. The genetic predisposition and immune system represent two important factors that can affect the various classification methods. The most common symptoms that turn up and characterize a skin pathological condition, include pain which is manifested as stinging and/or burning, itch that may be sporadic or persistent, localized or generalized, as well as functional disability (Mphande, 2020).

### Dioscorides and “*De Materia Medica*”

Over the last decades, research on medicinal plants has increasingly focused on the study of historical medico-botanical texts to identify plant species for further drug discovery and to comprehend the development of modern pharmacopoeias (Touwaide, 1992; Buenz et al., 2005; Leonti et al., 2010; Adams et al., 2011; Dal Cero et al., 2014). As a case in point, it is widely acknowledged that Dioscorides’ *De Materia Medica* has influenced and guided the development of Mediterranean and European traditional herbal medicine (Gurib-Fakim, 2006). Pedanius Dioscorides was born in Anazabra in the Cilicia Region of Anatolia in the first century A.D. It is known that he was a military physician in the Roman Army who travelled extensively in order to seek and explore medicinal substances to treat various ailments including skin diseases (Yildirim, 2013). Between AD 50 and 70, Dioscorides wrote his fundamental work that consists of a five-volume book in his native Greek, Περὶ ὕλης ἰατρικῆς (Peri hyles iatrikēs), known in Latin as *De Materia Medica*. Among many Greek manuscripts and texts, *De Materia Medica*, became the precursor to all modern pharmacopeias and transmitted the idea that investigation and experimentation performs a crucial role for pharmacology (Rooney, 2012). *De Materia Medica* is the most important text of botany and pharmacognosy, as well as the most detailed pharmacognostic guide that passed down from the ancient Mediterranean world, representing the prime historical source of information about the medicines used by the Greeks, Romans and other ethnic groups of antiquity. *De Materia Medica* incorporates 800 chapters in which Dioscorides monographed 600 different kinds of plants, 35 animals, and 90 minerals, summarizing the quintessence of medicinal remedies. Moreover, it includes detailed information about those drugs, such as their medical activities, methods of administration, habitat and methods of cultivation, botanical descriptions also illustrated by plant drawings, contraindications, dosages, veterinary, and non-medical uses (Gunther, 1968). In addition, Dioscorides drew on previous writings, his own experience as a physician as well as on local traditions in the Mediterranean and the Near East. Based on geographical references in the text, Dioscorides’ compilation is thought to be the fruit of extensive journeys while the predominant but contentious view is that he travelled extensively throughout Anatolia, Egypt, Arabia, Persia, Gallia, North Africa, and Caucasia (Staub et al., 2016). *De Materia Medica* is the most comprehensive and systematic work on simple drugs. It was translated into Syriac, Arabic, and Persian, as well as Latin and manually copied along with the botanical illustrations. It served as a corner stone for both western and eastern pharmaceutical and herbal knowledge, exerting a profound influence on the development of medicine in the Near East as well as in Europe. *De Materia Medica* of Dioscorides was closely and extensively studied by many medical writers and doctors of the Eastern and Western cultures. That is justified by the fact that the herbal remedies of Pedanius Dioscorides were transmitted to mediaeval Europe and the special characteristics of Arabic therapy was the widespread employment of drugs of all kinds (Yildirim, 2013). During the Middle Ages, the manual copies became more stylized and started to differ from the original botanical illustrations so, at the present time, the certainty about the accuracy of some species is diminished, hence the suggestions concerning the plant species described (Gaur et al., 2021). The information obtained by Dioscorides’ manuscript have undoubtedly influenced the traditional medical practices of the Balkans and the Mediterranean basin from the aspect of medicinal plants usage for the treatment of various skin diseases.

### Background History of the Study Area

Albania, Cyprus, Greece, and Turkey have long-standing historical and cultural ties linked to their geographical position, constant presence of their communities in Eastern Mediterranean, trade, and population movements. They share relations since antiquity, however this review is focused on the development of distinct medicinal plants commonly used against various skin ailments from the time of Dioscorides (*i.e.,* the Roman Empire), through the Byzantine and the Ottoman Empire, to modern era. After the fall of Roman Empire, Eastern Mediterranean region was under the control of Byzantium. During these centuries, medicinal plants’ therapeutic value was enriched by Arab herbal medicine, evolved, developed and preserved mainly through the transcription of herbals and codices by monks in monasteries (Isiksal, 2005; Azaizeh et al., 2006; Pan et al., 2014). The Ottoman Empire specifically at its peak in the 16th and 17th centuries CE, controlled not only southwestern Europe, mainland Greece, and the Balkans, but also parts of northern Iraq, Azerbaijan, Syria, Palestine, parts of the Arabian Peninsula, Egypt, and parts of the North African strip, in addition to the major Mediterranean islands of Rhodes, Cyprus, and Crete (Khan, 2020). Under the Ottoman rule the populations coexisted and lived through their Byzantine heritage and were solidly influenced by each other regarding cultural issues including healing techniques and medicinal remedies. It is important to underline the interdependence of Cyprus, Albania, and Turkey with the Greek customs and traditions. In addition, the island of Cyprus was mainly part of the Byzantium, the Eastern Roman Empire. After the fall of Rome, the knowledge of Greek medicine survived in the Byzantium and during the times of the Ottoman Empire many Greek Orthodox monasteries featured well-organized hospitals of the Byzantine traditions. These hospitals employed pharmacists to gather medicinal plants and prepare remedies, originating from Greek folk medicinal practices (Littlewood et al., 2002). The only extensive manuscript of local origin in this respect, is “Iatrosophikon,” which is a monastic scripture from the Ottoman period that contains prescriptions written down by the monk Mitrophanous (1790–1867) at the Greek Orthodox monastery of Makhairas in Cyprus (Lardos, 2006). Another historical highlight related to the modern history of Greece and Albania that represents the base of the Greek-Albanian relationship is “Northern Epirus,” the status of the Greek minority in Albania (Dervishi, 2019). Northern Epirus is a term used to refer to those parts of the historical region of Epirus, in the western Balkans, which today are part of Albania. The term is used mostly by Greeks and is associated with the existence of a substantial ethnic Greek population in the region (Smith and Hurst, 1999). This population, which is present in the Albanian territory until nowadays, supports the interconnection of the two countries and continues the past cultural exchange. Moreover, during the 17th-19th centuries, Epirus became the most famous center of folk medicine in the Balkan Peninsula. In an environment of economic affluence accompanied by an impressive cultural and intellectual life, the art of herbal healing developed and flourished. The medicine practitioners of the area were called “Vikoyiatri” which means doctors that come from Vikos gorge, a mountainous area situated in Epirus (Vokou et al., 1993). During spring and summer, they used to travel all over the Balkans, up to Istanbul (Constantinople during the Byzantine times), Bulgaria, Romania, and Russia, while even the Sultan or other Turkish officers asked for their advice or help (Vokou et al., 1993). However, at the end of the 19th century with the introduction of “western drugs” in the pharmacopoeias they were considered as charlatans and their invaluable knowledge on herbal medicine faded away. The first official pharmacopoeia of the newly formed Greek state (1830) was written in Greek and Latin by Vouros I., Landerer X.J., and Sartori J. in 1837, and it was mainly a translation of the Bavarian one. Earlier efforts, including the General Pharmacopeia based on scripts of Dionysios Pyrros of Thessaly published in Istanbul by Brugnatelli in 1818, were not officially recognized. In 1831, Dionysios Pyrros published additionally a two-volume medical guide in which he described 450 medicines and 150 medicinal plants for the treatment of 362 ailments. Likewise, no recognition was made for the “Greek Pharmacopoeia” by Foteinos G., published in Ismir, Turkey, in 1835 (Karabelopoulos et al., 2004).

## Methods

**FIGURE 1:**
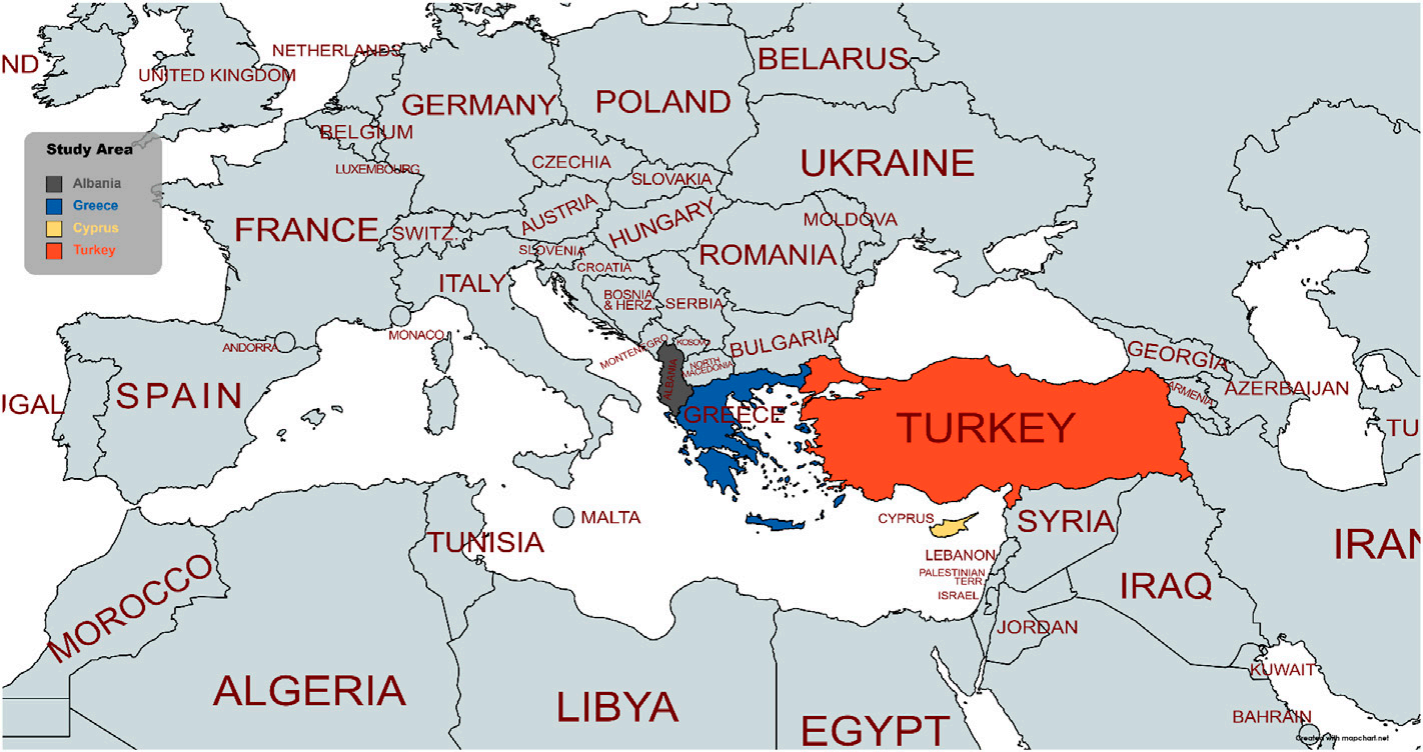
Map of the study area.

Some of the most important scientific databases such as Scopus, PubMed, ScienceDirect, and Google scholar were browsed to perform a literature search in order to identify all the published ethnobotanical field studies conducted in Albania, Cyprus, Greece, and Turkey (Figure 1) up until May 2020. The search was carried out by employing specific keywords or their combinations. The keywords used were “ethnobotanical,” “ethnobotany,” “ethnopharmacological,” “ethnopharmacology,” “ethnomedicinal,” and “ethnomedicine,” followed by the word “Balkans” or the name of each country studied. Only published field studies that included interviews with informants were considered, so published reviews such as the important work of Jarić et al. (2018) or the study of *Iatrosofikon* manuscript by Lardos (2006) were excluded from this review. Through the extensive literature search, data concerning 128 published ethnobotanical field studies were found and elaborated. Most of the studies (Paksoy et al., 2016) concerned traditional medicine in Turkey, 14 studies referred to Greece, 7 studies to Albania and 5 studies to Cyprus. The data relative to plant uses against skin disorders were manually retrieved from each study and recorded as multiple entries in an Excel file (.xlsx format). Afterwards, data for each species were merged in a single row with multiple columns including the botanical name, the vernacular name, the family, the country, and the region where the ethnobotanical study has been conducted, the plant part used, the preparation form with eventual details in case of a recipe and the ailments treated or the therapeutic effects. The skin diseases extracted from the publications were summarized and classified based on the terminology used in dermatology and grouped in 37 different categories (Table 1). In order to facilitate the data elaboration, plant subspecies were clustered with their corresponding species, when applicable. In addition, the botanical names of the plants reported were validated through the databases “The Plant List” (The Plant List [Internet], 2013) and “The Global Biodiversity Information Facility” (GBIF.org [Internet], 2020). If the original plant name from the references is a synonym of an accepted species, it is mentioned in parenthesis e.g., *Centaurea cyanus* L. (synonym of *Cyanus segetum* Hill), where *Cyanus segetum* Hill is the accepted species and *Centaurea cyanus* L. the synonym. Furthermore, synonyms of an accepted species, that was already reported in a study, are also mentioned in parenthesis, e.g., *Allium ampeloprasum* L. (= *Allium porrum* L.). Data curation and statistical analysis was performed in EXCEL.

**TABLE 1:** Skin diseases extracted from literature data and grouped in 37 categories in alphabetical order.

Skin diseases	Ailment group
Acne, Pimples, Over production of sebum, Oily skin	G1
Alopecia, Hair loss, Baldness, Hair follicle stimulator, Hair loss prevention	G2
Anti-bleeding, Hemostatic, Blood stopper, Nose bleeding, Antihemorrhagic, Epistaxis	G3
Antifungal, Antibacterial, Dermatophyte, Mycodermatitis, Antiseptic, Disinfectant, Mycosis, Fumigant, Germicidal, Cleaning of the foulness of ulcers	G4
Anti-inflammatory	G5
Aphthae, Stomatitis, Mouth sores	G6
Blisters, Vesicle	G7
Body itch, Urticaria, Prickly Heat, Pruritus	G8
Boils, Abscess, Carbuncle, Furuncles, Ingrown hair, Inflamed wound, Fistulas, Felon	G9
Bruises, Contusions, Ecchymosis, Purpleness	G10
Callouses	G11
Cellulites	G12
Chloasma, Skin lightener, Freckles, Vitiligo, Pigmentation	G13
Dandruff	G14
Depilatory	G15
Dog bite, Snake bite, Insect stings, Bee bite	G16
Eczema	G17
Emollient, Moisturizer	G18
Erysipelas	G19
Excrescences (warts, raised moles), Verruca, Moles, Skin Tumors	G20
Gout	G21
Hemorrhoids, Piles	G22
Herpes, Papilloma	G23
Keratolytic	G24
Lice infestation, Pediculosis, Parasitical skin diseases	G25
Peeling of facial skin, Flaking of facial skin, Exfoliation	G26
Psoriasis	G27
Rash, Facial skin eruption, Erythema, Intertrigo	G28
Ringworm, Lichens	G29
Scabies	G30
Scars, Stretch marks, Blemishes	G31
Skin ailments, Skin diseases, Skin disorders (undefined)	G32
Sores, Trauma, Injury, Wounds (burn wound, septic wounds, festering wounds), Fissure, Chapped, Cracks, Scorch, Lesions, Cleft, Cutaneous eruption, Scalds, Kibes, Vulnerary, Cicatrizing	G33
Styptic, Astringent	G34
Sweat	G35
Whitlow, Swelling, Edema	G36
Wrinkled skin	G37
Leprosy^a^	G38

a Reported only in *De Materia Medica*.

## Results and Discussion

### Plant Species Reported in Ethnobotanical Research of the Study Area

**FIGURE 2:**
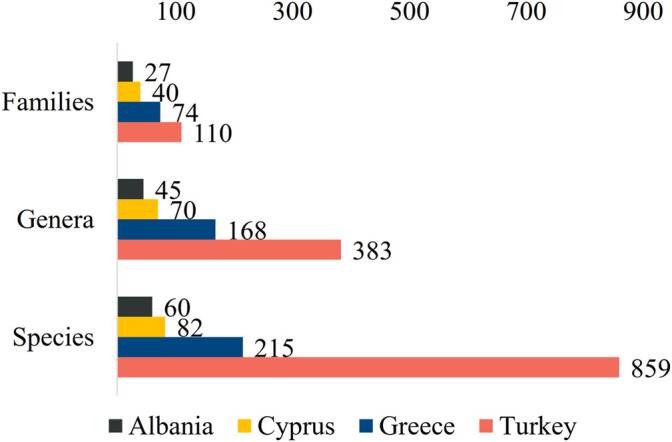
Total reports of families, genera, and species per country.

The bibliographical analysis indicated a total of 967 taxa belonging to 418 different genera and 111 different families that were used against skin related diseases. Specifically, 27 different families are reported in Albania, 40 in Cyprus, 74 in Greece, and 110 in Turkey (Figure 2).

**FIGURE 3:**
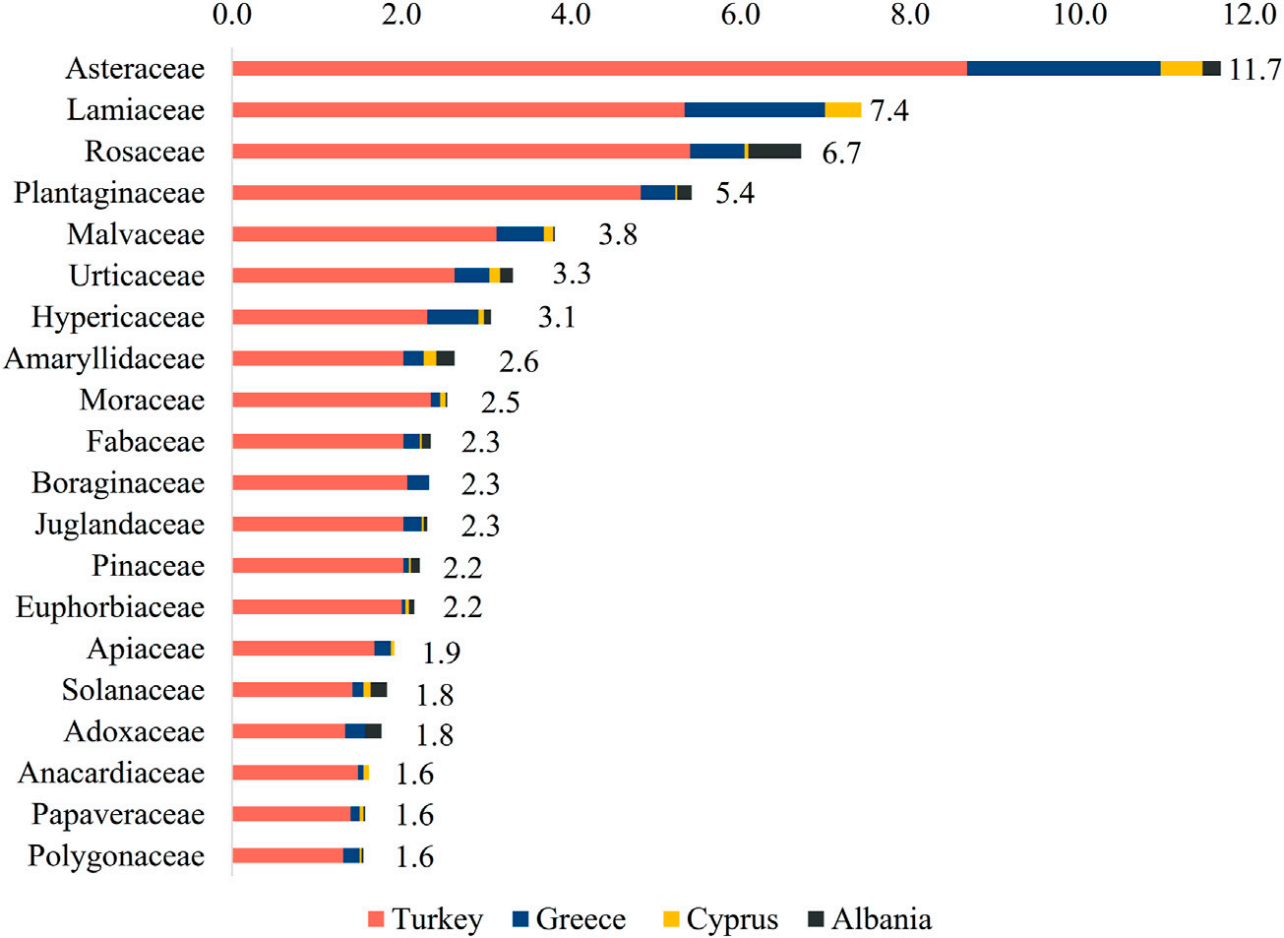
Most cited families in relation to skin ailment reports.

Out of 111 different families reported, the families mostly cited were Asteraceae (542 uses, 11.7%), Lamiaceae (345 uses, 7.4%), Rosaceae (312 uses, 6.7%), Plantaginaceae (252 uses, 5.4%), Malvaceae (177 uses, 3.8%), Urticaceae (154 uses, 3.3%), Hypericaceae (142 uses, 3.1%), Moraceae (118 uses, 2.5%), Fabaceae (109 uses, 2.3%), Boraginaceae (108 uses, 2.3%), Juglandaceae (107 uses, 2.3%), Pinaceae (103 uses, 2.2%), Euphorbiaceae (100 uses, 2.2%), Apiaceae (89 uses, 1.9%), Solanaceae (85 uses, 1.8%), Adoxaceae (82 uses, 1.8%), Anacardiaceae (75 uses, 1.6%), Papaveraceae (73 uses, 1.6%), and Polygonaceae (72 uses, 1.6%). The families Lamiaceae, Apiaceae, and Anacardiaceae were reported in three of the four countries (Cyprus, Greece, and Turkey), as well as Adoxaceae (Albania, Greece, and Turkey), Boraginaceae only in two countries (Greece and Turkey), while the rest are present in ethnobotanical studies conducted in all four countries (Figure 3).

**FIGURE 4:**
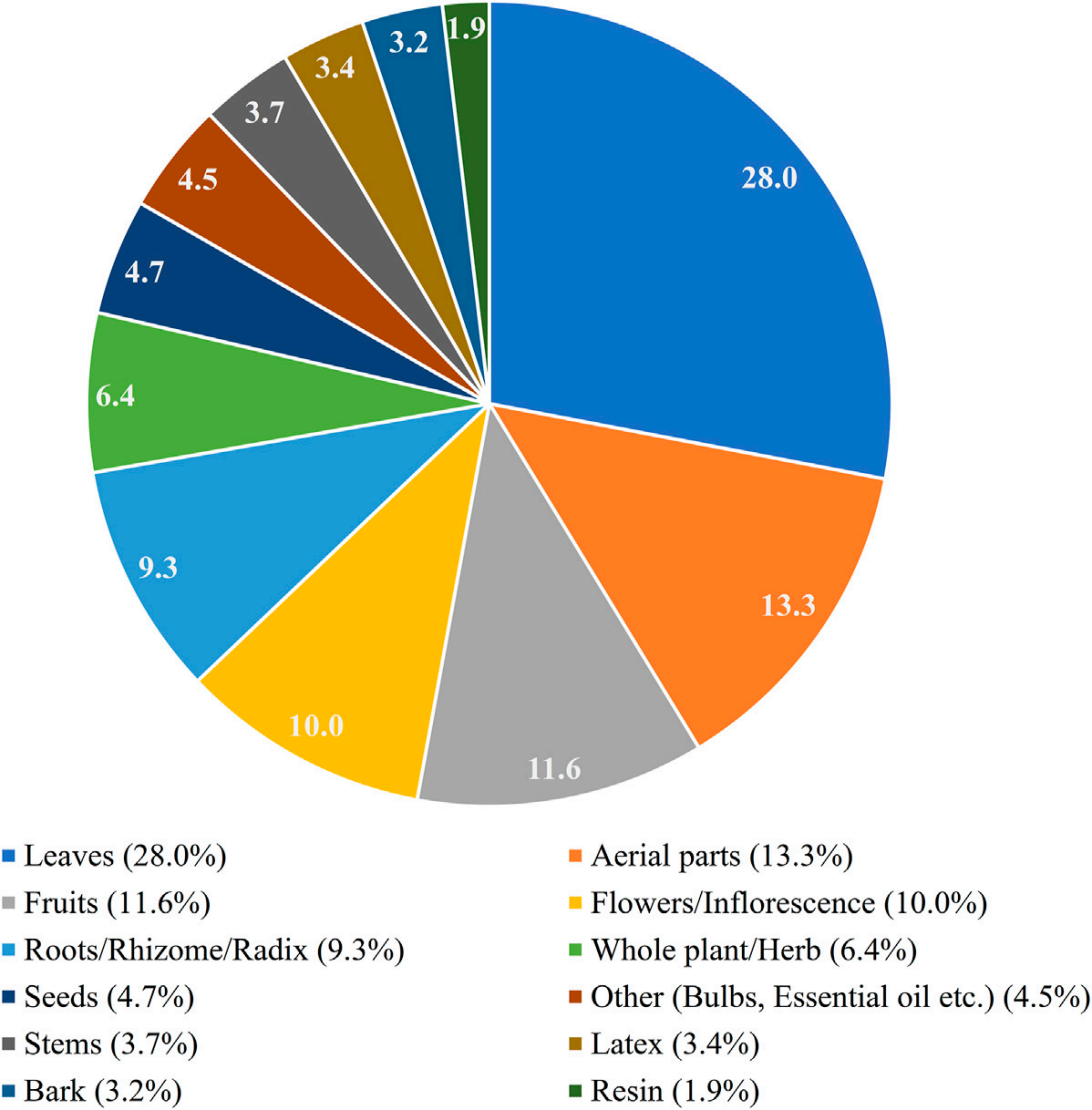
Most cited plant parts in relation to skin ailment reports.

Many different ways of preparation were reported. The most cited ones were decoction or infusion, taken as a drink or used externally. Other methods reported were using plants to prepare a poultice, an ointment, a compress, or just using the plant externally. A total of 3,947 reports on plant parts were reported. The most cited plant parts used were the leaves (1105 reports , 28.0%), the aerial parts (525 reports, 13.1%), the fruits (457 reports, 11.6%), the flowers/inflorescence (396 reports, 10.0%), the roots/rhizome/radix (369 reports, 9.3%), the whole space plant/herb (252 reports, 6.4%), the seeds (184 reports, 4.7%),the stems (148 reports, 3.7%), the latex (133 reports, 3.4%), the bark (128 reports, 3.2%), and the resin (74 reports, 1.9%). Other parts used, including bulbs and essential oils had 176 reports (4.5%) (Figure 4.)

**FIGURE 5:**
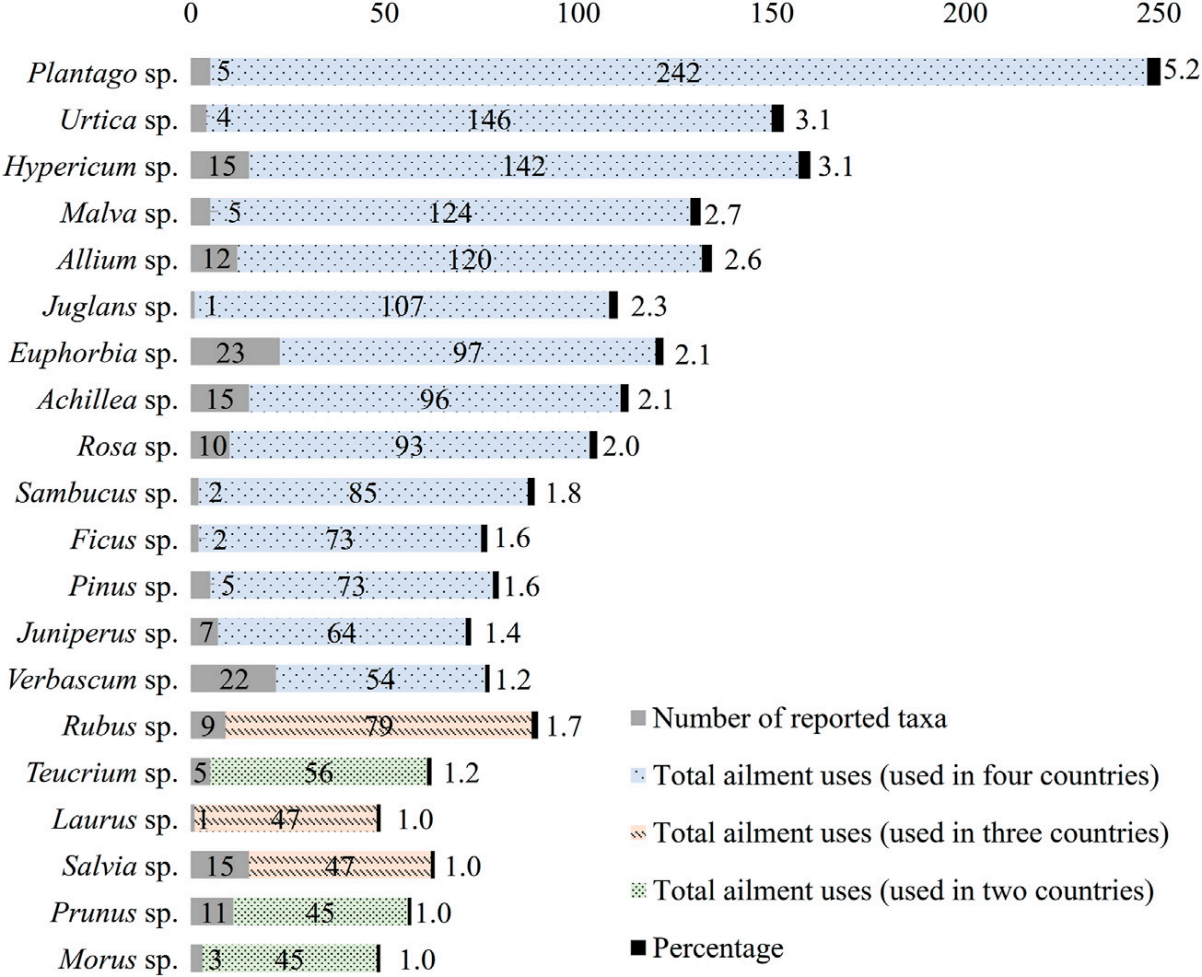
Most cited genera in relation with their corresponding taxa.

In Figure 5 the most cited genera in relation to their total use in skin related diseases are shown, along with the number of reported taxa of the same genus. These are *Plantago* L. sp. (5 taxa, 242 uses, 5.2%), *Urtica* L. sp. (4 taxa, 146 uses, 3.1%), *Hypericum* L. sp. (15 taxa, 142 uses, 3.1%), *Malva* L sp. (5 taxa, 124 uses, 2.7%), *Allium* L. sp. (12 taxa, 120 uses, 2.6%), *Juglans* L. sp. (1 taxon, 107 uses, 2.3%), *Euphorbia* sp. (23 taxa, 97 uses, 2.1%), *Achillea* L. sp. (15 taxa, 96 uses, 2.1%), *Rosa* L. sp. (10 taxa, 93 uses, 2.0%), *Sambucus* sp. (2 taxa, 85 uses, 1.8%), *Ficus* L. sp. (2 taxa, 73 uses, 1.6%), *Pinus* L. sp. (5 taxa, 73 uses, 1.6%), *Juniperus* L. sp. (7 taxa, 64 uses, 1.4%), *Verbascum* L. sp. (22 taxa, 54 uses, 1.2%), *Rubus* L sp. (9 taxa, 79 uses, 1.7%), *Teucrium* L. sp. (5 taxa, 56 uses, 1.2%), *Laurus* L. sp. (1 taxon, 47 uses, 1.0%), *Salvia* L. sp. (15 taxa, 47 uses, 1.0%), *Prunus* L. sp. (11 taxa, 45 uses, 1.0%), and *Morus* L. sp. (3 taxa, 45 uses, 1.0%). Genera *Teucrium* L. and *Morus* L. were reported in only two countries, Greece and Turkey, as well as *Prunus* L. sp. that was reported only in Albania and Turkey. *Rubus* L. sp. was reported in three of the countries (Albania, Greece, and Turkey)*,* as well as *Laurus* L. sp. and *Salvia* L. sp. (Cyprus, Greece, and Turkey), while the rest were reported in ethnobotanical studies conducted in all four countries. Specifically, 45 different genera are reported in Albania, 70 in Cyprus, 168 in Greece, and 383 in Turkey.

**FIGURE 6:**
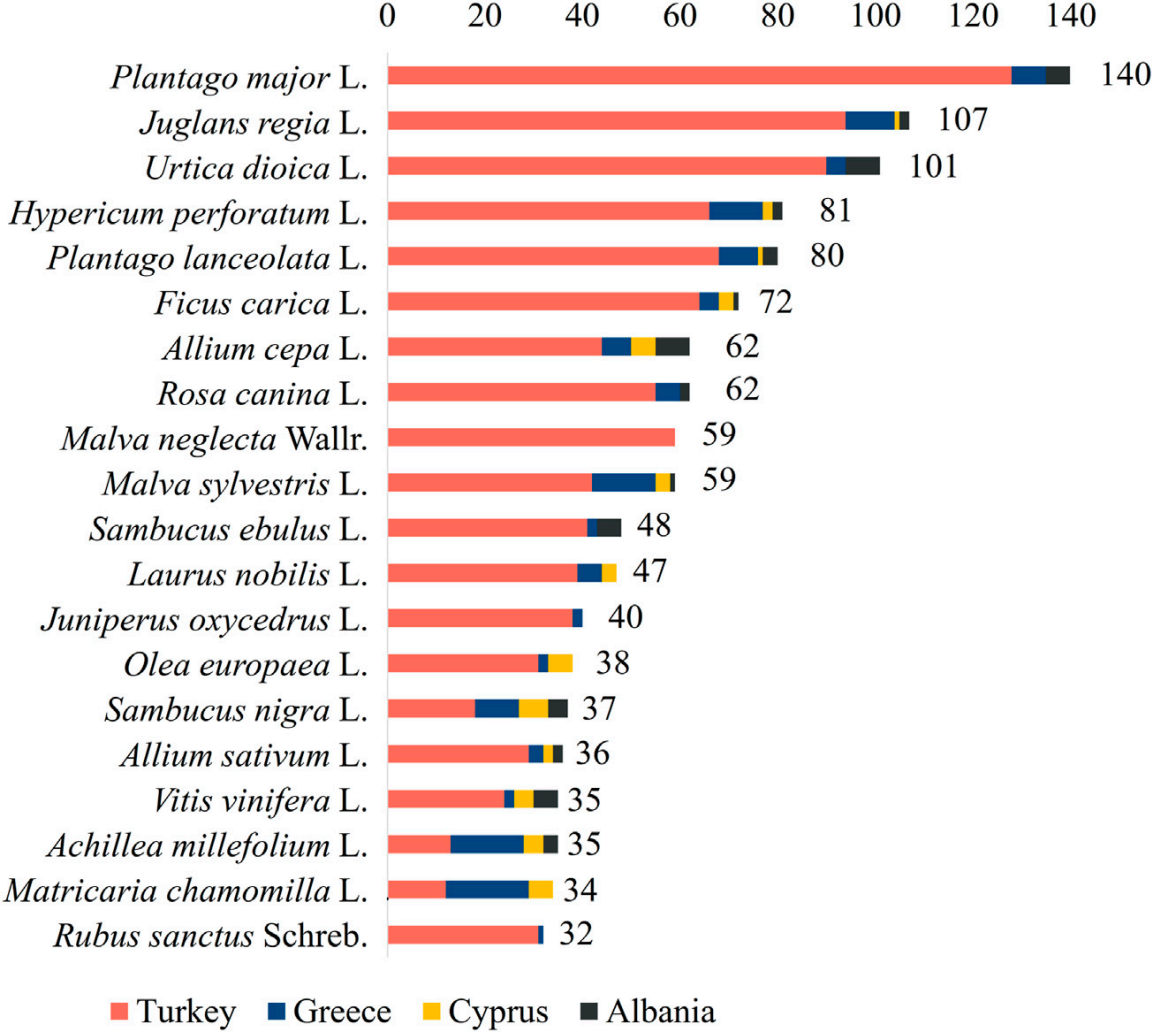
Most cited plant species in relation to skin ailment reports.

The most cited plants species used for the treatment of skin ailments were *Plantago major* L. (140 uses, 3.0%), *Juglans regia* L. (107 uses, 2.3%), *Urtica dioica* L. (101 uses, 2.2%), *Hypericum perforatum* L. (81 uses, 1.7%), *Plantago lanceolata* L. (80 uses, 1.7%), *Ficus carica* L. (72 uses, 1.6%), *Allium cepa* L. (62 uses, 1.3%), *Rosa canina* L. (62 uses, 1.3%), *Malva neglecta* Wallr. (59 uses, 1.3%), *Malva sylvestris* L. (59 uses, 1.3%), *Sambucus ebulus* L. (48 uses, 1.0%), *Laurus nobilis* L. (47 uses, 1.0%), *Juniperus oxycedrus* L. (40 uses, 0.9%), *Olea europaea* L. (39 uses, 0.8%), *Sambucus nigra* L. (37 uses, 0.8%), *Allium sativum* L. (36 uses, 0.8%), *Vitis vinifera* L. (35 uses, 0.8%), *Achillea millefolium* L. (35 uses, 0.8%), *Matricaria chamomilla* L. (34 uses, 0.7%), and *Rubus sanctus* Schreb. (32 uses, 0.7%). It is important to underline that *P. major*, *U. dioica*, *R. canina,* and *S. ebulus* were reported in ethnobotanical studies in three of the four countries of the study area (Albania, Greece, and Turkey), as well as *L. nobilis*, and *M. chamomilla* (Cyprus, Greece, and Turkey). *J. oxycedrus* and *R. sanctus* were reported in ethnobotanical studies in two countries (Greece and Turkey), *M. neglecta* was reported only in Turkey, while the rest of the plants are used in all four countries (Figure 6).

**FIGURE 7:**
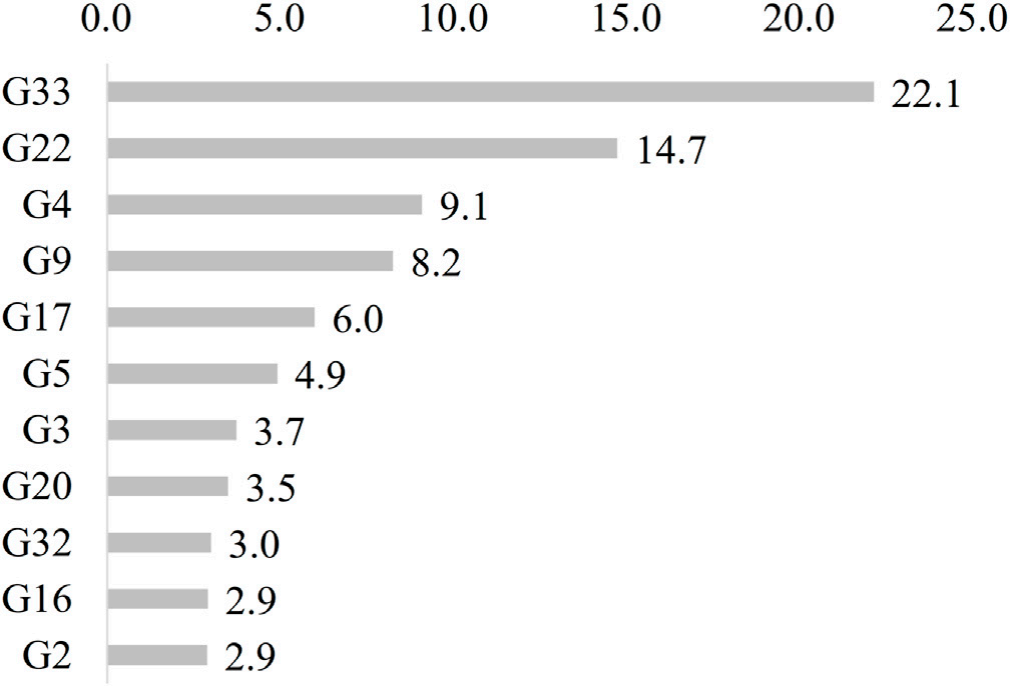
Most cited ailment categories (G33 is Wounds etc., G22 is Hemorrhoids etc., G4 is Antibacterial etc., G9 is Boils etc., G17 is Eczema, G5 is Anti-inflammatory, G20 is Excrescences etc., G3 is Antibleeding etc., G32 is General skin ailments, and G16 is Dog bites etc.).

A total of 4,645 reports of skin related ailments were catalogued. The most cited categories identified in the studies were wounds etc. (G33, 1028 reports, 22.1%), hemorrhoids etc. (G22, 684 reports, 14.7%), antibacterial etc. (G4, 422 reports, 9.1%), boils etc. (G9, 383 reports, 8.2%), eczema (G17, 278 reports, 6.0%), anti-inflammatory (G5, 228 reports, 4.9%), antibleeding etc. (G3, 173 reports, 3.7%), excrescences etc. (G20, 162 reports, 3.5%), general skin ailments (G32, 139 reports, 3.0%), dog bites etc. (G16, 135 reports, 2.9%), and alopecia etc (G2, 134 reports, 2.9%) (Figure 7).

**FIGURE 8:**
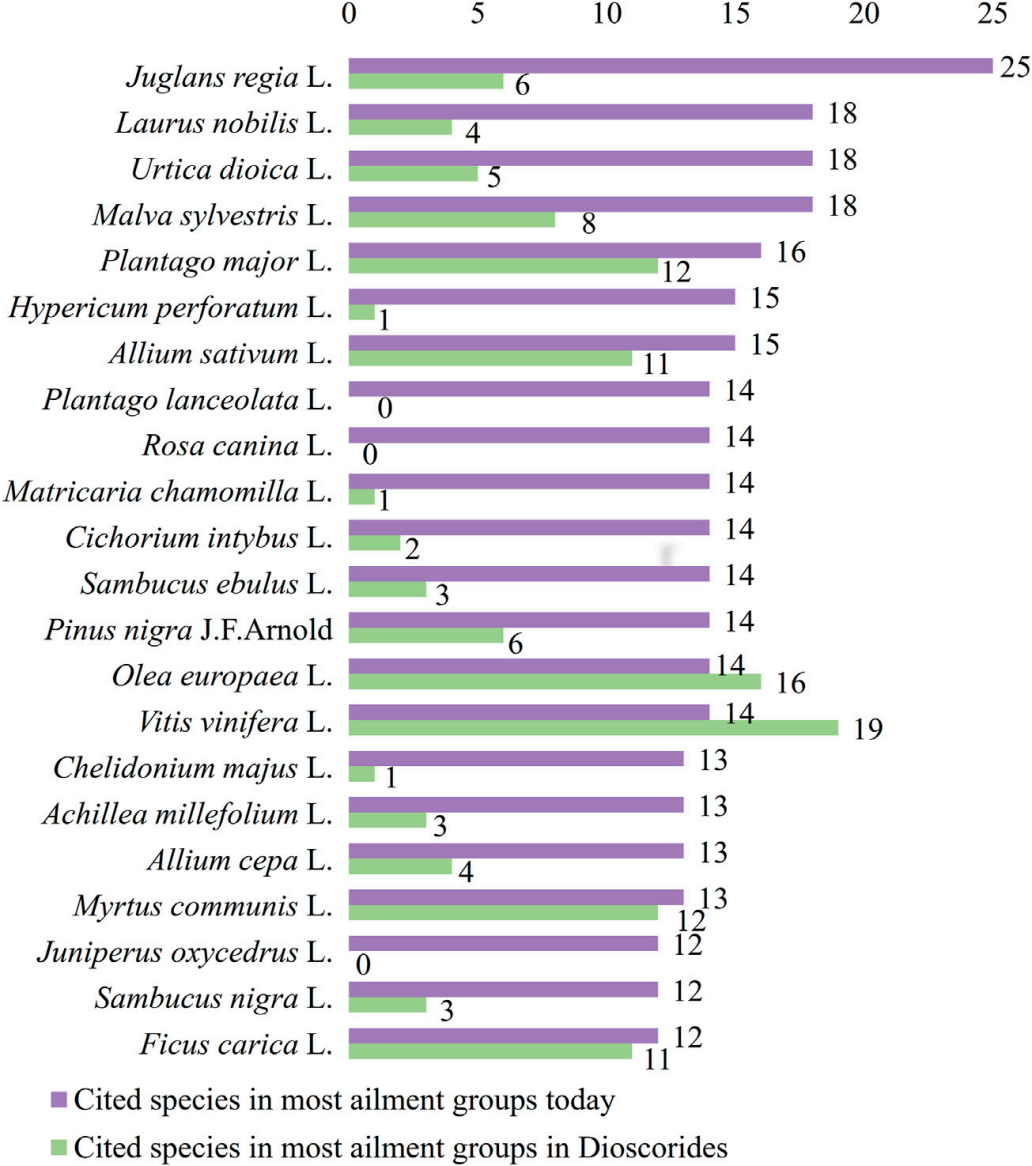
Cited species in most ailment groups. Comparison between contemporary data and Dioscorides.

Finally, out of 37 different skin ailment groups, the plants used for the treatment of most of them were *J. regia* (25 different skin ailment groups), *L. nobilis* (18 groups), *M. sylvestris* (18 groups), *U. dioica* (18 groups), *P. major* (16 groups), *A. sativum* (15 groups), *H. perforatum* (15 groups), *Cichorium intybus* L. (14 groups), *M. chamomilla* (14groups), *O. europaea* (14 groups), *P. nigra* (14 groups), *P. lanceolata* (14 groups), *R. canina* (14 groups), *S. ebulus* (14 groups), *V. vinifera* (14 groups), *A. millefolium* (13 groups), *A. cepa* (13 groups), *Chelidonium majus* L. (13 groups), *Myrtus communis* L. (13 groups), *F. carica* (12 groups), *J. oxycedrus* (12 groups), and *S. nigra* (12 groups). Most of these plants comprise the most cited plants as well, with the exception of *P. nigra* which was reported only in Turkey, *C. intybus* which was reported in Greece and Turkey and *H. perforatum* and *C. majus* which were reported in ethnobotanical studies in all four countries (Figure 8).

### Plant Species Reported in Dioscorides “*De Materia Medica*”

The extensive study of Dioscorides’ manuscript, translated in English by Osbaldeston and Wood (Dioscorides et al., 2000) led to the discovery of 289 different entries in respect of treatments against skin related problems. Each entry contained suggested modern botanical names for the plants described by Dioscorides. The suggested plant names reported in each entry were validated by the databases and were eventually consolidated into 275 different entries, since several entries corresponded to the same plant species. The method of cataloguing each entry was performed in the same way as in the analysis of the field studies described above.

**FIGURE 9:**
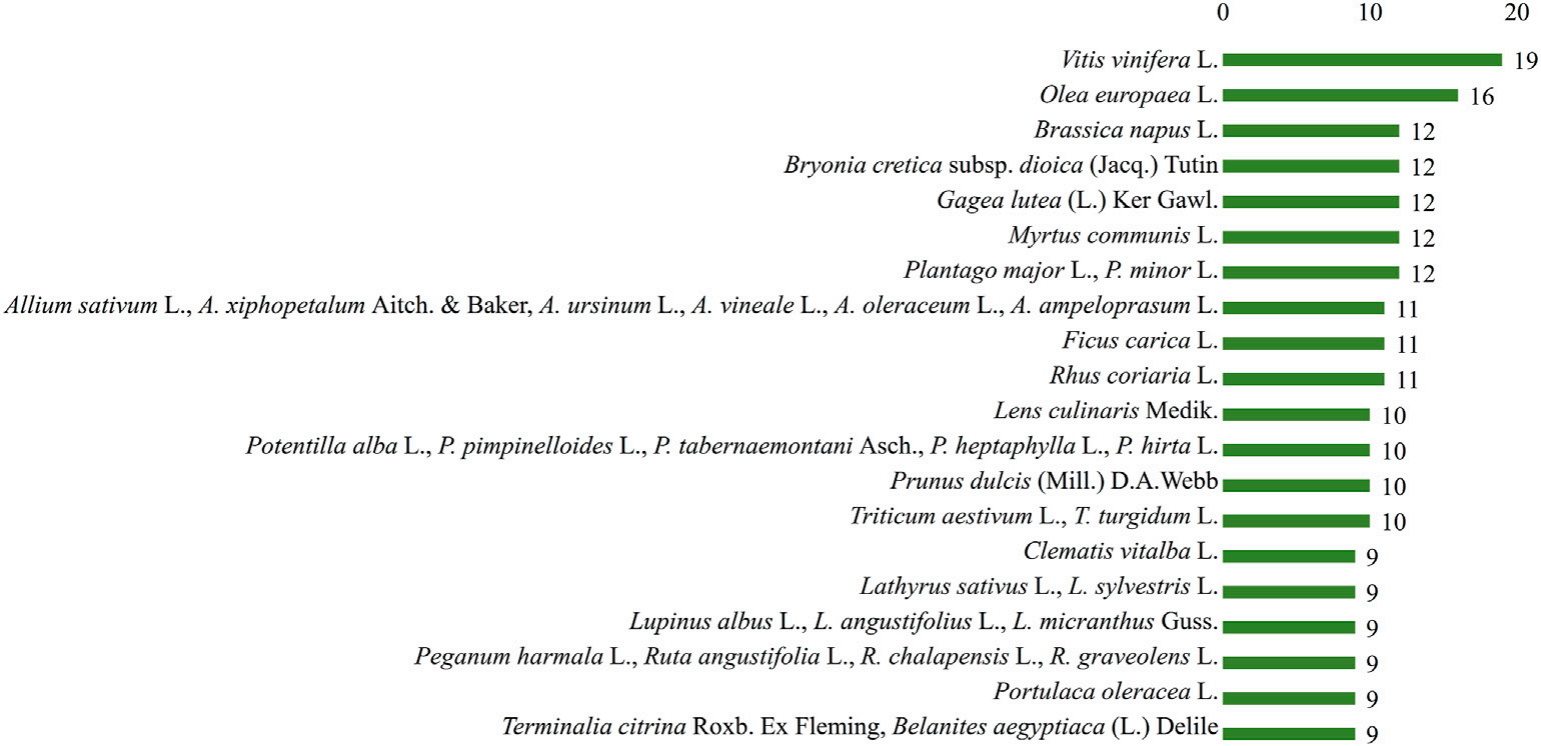
Suggested species with the highest number of reported uses according to Dioscorides.

The suggested species with the highest number of reported uses among all the aliment categories (Figure 9) were *V. vinifera* L. (19 groups), *O. europaea* L. (16 groups), *Brassica napus* L. (12 groups), *Bryonia cretica* subsp. *dioica* (Jacq.) Tutin (12 groups), *Gagea lutea* (L.) Ker Gawl. (12 groups), *M. communis* L. (12 groups), *P. major* L., *P. minor* L.) (12 groups), *A. sativum* L., *Allium xiphopetalum* Aitch. & Baker, *Allium ursinum* L., *Allium vineale* L., *Allium oleraceum* L., *Allium ampeloprasum* L.) (11 groups), *F. carica* L. (11 groups), *Rhus coriaria* L. (11 groups), *Lens culinaris* Medik. (10 groups), *Potentilla alba* L., *Potentilla pimpinelloides* L., *Potentilla tabernaemontani* Asch., *Potentilla heptaphylla* L., *Potentilla hirta* L. (10 groups), *Prunus dulcis* (Mill.) D.A.Webb (10 groups), *Triticum aesetivum* L., *Triticum turgidum* L.) (10 groups), *Clematis vitalba* L. (9 groups), *Lathyrus sativus* L*., Lathyrus sylvestris* L. (9 groups), *Lupinus albus* L., *Lupinus angustifolius* L., *Lupinus micranthus* Guss. (9 groups), *Peganum harmala* L., *Ruta angustifolia* Pers., *Ruta chalepensis* L., *Ruta graveolens* L. (9 groups), *Portulaca oleracea* L. (9 groups), and *Terminalia citrina* Roxb. ex Fleming, *Balanites aegyptiaca* (L.) Delile (9 groups), while the preparation methods were similar to the ones used today.

The skin ailments described in *De Materia Medica* were also clustered in 37 different groups (Table 1), with the addition of the category “Leprosy” (G38), in order to obtain a better comparison with the skin ailment groups described in modern ethnobotanical field studies. The lack of data concerning Leprosy (G38) in the modern ethnobotanical studies can be attributed to the fact that leprosy greatly diminished in the study area around 1960 (Kyriakis et al., 1994; Lechat et al., 2002; Reibel et al., 2015).

**FIGURE 10:**
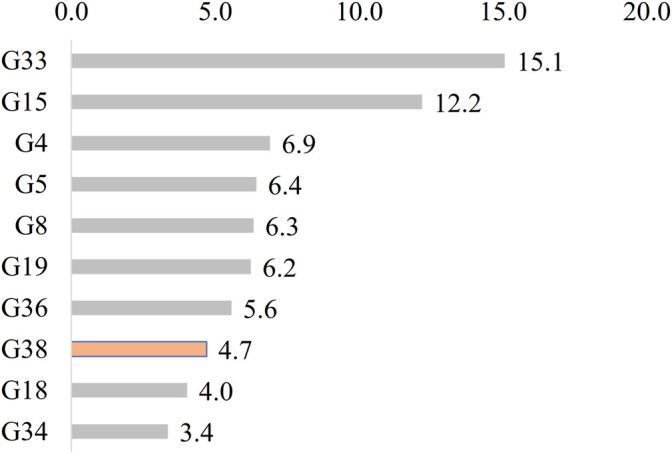
Most cited ailment categories according to Dioscorides. Leprosy (G38) is only reported in Dioscorides.

A total 1,042 reports were recorded. In Figure 10, the most cited ailment categories treated according to Dioscorides are shown. These are wounds etc. (G33, 157 reports 15.1%), dog bites etc. (G15, 127 reports, 12.2%), antibacterial etc. (G4, 72 reports, 6.9%), anti-inflammatory (G5, 67 reports, 6.4%), boils etc. (G8, 66 reports, 6.3%), excrescences etc. (G19, 65 reports, 6.2%), whitlow etc. (G36, 58 reports, 5.6%), leprosy (G38, 49 reports, 4.7%) which is highlighted in the chart, erysipelas (G18, 42 reports, 4.0%), and styptic (G34, 35 reports, 3.4%). Three groups, such as cellulites (G11), keratolysis (G23) and general skin ailments (undefined) (G32), are not mentioned in *De Materia Medica*.

It is important to mention that 19 of the 22 most reported plants used for the treatment of most of the skin ailment groups used in traditional medicine in the study area are also present in Dioscorides’ *De Materia Medica* and employed for the treatment of some of these (Figure 8). These are *J. regia* L. (G2, G4, G5, G9, G16, and G33), *L. nobilis* L. (G5 and G16), *U. dioica* L. (G4, G9, G16, and G33), *M. sylvestris* L. (G4, G5, G6, G16, G33, and G34), *P. major* L. (G3, G4, G5, G9, G16, G20, G23, and G33), *H. perforatum* L. (G33), *A. sativum* L. (G2, G10, G16, G29, and G33), *M. chamomilla* L. (G6), *S. ebulus* L. (G5, G16, and G33), *P. nigra* J.F.Arnold (G2, G4, G9, G16, and G33), *O. europaea* L. (G4, G5, G6, G9, G20, G33, and G36), *V. vinifera* L. (G4, G5, G6, G9, G10, G14, G16, G25, G29, and G33), *C. majus* L. (G23), *A. millefolium* L. (G3, G9, and G33), *A. cepa* L. (G2, G9, and G16), *M. communis* L. (G1, G2, G9, G28, and G33), *S. nigra* L. (G5 and G33), and *F. carica* L. (G4, G9, G16, G20, and G33). Two plant species are not mentioned in the ancient manuscript (*P. lanceolata* L. and *J. oxycedrus* L.), one species (*C. intybus* L.) is mentioned but not for the same skin ailments, while one species (*R. canina* L.) is mentioned, but not for skin related diseases (Table 2).

**TABLE 2:** Species used in most ailment categories in the contemporary ethnobotanical studies which are also reported for the same uses in Dioscorides’ manuscript.

Plant species	Common ailment categories
*Juglans regia* L.	G2, G4, G5, G9, G16, G33
*Laurus nobilis* L.	G5, G16
*Urtica dioica* L.	G4, G9, G16, G33
*Malva sylvestris* L.	G4, G5, G6, G16, G33, G34
*Plantago major* L.	G3, G4, G5, G9, G16, G20, G23, G33
*Hypericum perforatum* L.	G33
*Allium sativum* L.	G2, G10, G16, G29, G33
*Plantago lanceolata* L.	Not mentioned in Dioscorides
*Rosa canina* L.	No skin related uses reported in Dioscorides
*Matricaria chamomilla* L. (= *Matricaria recutita* L.)	G6
*Cichorium intybus* L.	Mentioned in Dioscorides, but not for the same skin ailments
*Sambucus ebulus* L.	G5, G16, G33
*Pinus nigra* J.F.Arnold	G2, G4, G9, G16, G33
*Olea europaea* L.	G4, G5, G6, G9, G20, G33, G36
*Vitis vinifera* L. (= *Vitis sylvestris* C.C.Gmel.)	G4, G5, G6, G9, G10, G14, G16, G25, G29, G33
*Chelidonium majus* L.	G23
*Achillea millefolium* L.	G3, G9, G33
*Allium cepa* L.	G2, G9, G16
*Myrtus communis* L.	G1, G2, G9, G28, G33
*Juniperus oxycedrus* L.	Not mentioned in Dioscorides
*Sambucus nigra* L.	G5, G33
*Ficus carica* L.	G4, G9, G16, G20, G33

The results obtained during the extensive bibliographical analysis of the ethnobotanical field studies are presented in Table 3 in alphabetical order. Only 215 taxa used in traditional medicine in Greece are shown, along with their corresponding families. A comparison of the occurrence of these plants was also carried out between Greece, Albania, Cyprus, Turkey, and Dioscorides’ *De Materia Medica.* The number of their total uses against skin ailment categories in the study area was calculated.

**TABLE 3:** Taxa reported in Greek ethnobotanical field studies, cross-referenced with the other countries and *De Materia Medica*.

Botanical name	Family	Albania	Cyprus	Turkey	Skin Disease group (Nο. of occurrence) ±	References
*Abutilon theophrasti* Medik.	Malvaceae				G33 (1)	Axiotis et al. (2018)
^b^ *Achillea holosericea* Sm.	Asteraceae				G22 (1)	Tsioutsiou et al. (2019)
^a^ *Achillea millefolium* L.	Asteraceae	+	+	+	G1 (4), G2 (2), G3 (6), G4 (2), G5 (2), G9 (2), G17 (1), G22 (3), G26 (1), G28 (1), G32 (1), G33 (9), G34 (1)	Sezik et al. (1997); Brussell, (2004); Hanlidou et al. (2004); Ugulu et al. (2009); Çakilcioǧlu and Türkoğlu, (2009); Altundag and Ozturk, (2011); Karousou and Deirmentzoglou, (2011); Akgül et al. (2016); Pieroni, (2017); Pieroni and Sõukand, (2017); Tsioutsiou et al. (2017); Karakaya et al. (2019); Karakaya et al. (2020); Tsioutsiou et al. (2019); Petrakou et al. (2020)
^b^ *Acinos suaveolens* (Sm.) G. Don ex Loudon [synonym of *Clinopodium suaveolens* (Sm.) Kuntze]	Lamiaceae				G4 (1), G33 (1)	Hanlidou et al. (2004); Adamidou, (2012)
^a^ *Adiantum capillus-veneris* L.	Pteridaceae			+	G1 (1), G2 (2)	Hanlidou et al. (2004); Güzel et al. (2015)
*Aesculus hippocastanum* L.	Sapindaceae	+		+	G16 (1), G22 (2)	Ugulu et al. (2009); Pieroni et al. (2014a); Petrakou et al. (2020)
^a^ *Agrimonia eupatoria* L.	Rosaceae	+		+	G4 (2), G5 (2), G16 (1), G22 (1), G33 (4), G34 (2), G36 (1)	Everest and Ozturk, (2005); Pieroni, (2008); Adamidou, (2012); Tetik et al. (2013); Tsioutsiou et al. (2017); Axiotis et al. (2018); Gürbüz et al. (2019); Tsioutsiou et al. (2019); Petrakou et al. (2020)
^a^ *Ajuga reptans* L.	Lamiaceae				G5 (1), G33 (1)	Brussell, (2004)
*Alchemilla vulgaris* L.	Rosaceae				G3 (1)	Adamidou, (2012)
^a^ *Alkanna tinctoria* (L.) Tausch	Boraginaceae			+	G4 (1), G8 (1), G16 (1), G22 (3), G33 (3)	Ari et al. (2015); Güzel et al. (2015); Axiotis et al. (2018); Papageorgiou et al. (2020)
^a^ *Alliaria officinalis* Andrz. ex DC. [synonym of *Alliaria petiolata* (M.Bieb.) Cavara & Grande]	Brassicaceae				G17 (1)	Axiotis et al. (2018)
^a^ *Allium ampeloprasum* L. (= *Allium porrum* L.)	Amaryllidaceae	+		+	G4 (1), G22 (2), G33 (1)	Sezik et al. (2001); Pieroni et al. (2014a); Axiotis et al. (2018); Akbulut et al. (2019)
^a^ *Allium cepa* L.	Amaryllidaceae	+	+	+	G2 (1), G4 (3), G5 (3), G6 (1), G9 (16), G10 (12), G16 (1), G22 (2), G25 (1), G31 (2), G32 (1), G33 (15), G36 (4)	Malamas and Marselos, (1992); Fujita et al. (1995); Yeşilada et al. (1995); Sezik et al. (1997); Yeşilada et al. (1999); Sezik et al. (2001); Hanlidou et al. (2004); Pieroni et al. (2005a); Pieroni et al. (2005b); Everest and Ozturk, (2005); Ezer and Mumcu Arisan, (2006); Pieroni et al. (2006); Kültür, (2007); Kargioǧlu et al. (2008); Yildirim et al. (2008); Ugulu et al. (2009); Karakaya et al. (2020); Çakilcioǧlu and Türkoğlu, (2009); Yöney et al. (2010); Lardos and Heinrich, (2013); Pieroni et al. (2014a); Pieroni et al. (2014b); Hayta et al. (2014); Polat et al. (2015); Günbatan et al. (2016); Uzun and Kaya, (2016); Güneş et al. (2017); Karcı et al. (2017); Pieroni, (2017); Pieroni and Sõukand, (2017); Gürbüz et al. (2019); Tsioutsiou et al. (2019)
^a^ *Allium sativum* L.	Amaryllidaceae	+	+	+	G2 (6), G4 (4), G5 (2), G8 (1), G9 (1), G10 (1), G16 (5), G17 (1), G22 (5), G23 (1), G28 (1), G29 (4), G33 (2), G33 (1), G34 (1)	Akgül et al. (2016); Karakaya et al. (2019); Hanlidou et al. (2004); Pieroni and Sõukand, (2017); Çakilcioǧlu and Türkoğlu, (2009); Güzel et al. (2015); Gürbüz et al. (2019); Sezik et al. (2001); Pieroni et al. (2006); Pieroni et al. (2014b); Yöney et al. (2010); Yeşilada et al. (1995); Yeşilada et al. (1999); Ezer and Mumcu Arisan, (2006); Kültür, (2007); Hayta et al. (2014); Uzun and Kaya, (2016); Karaman and Kocabas, (2001); Gözüm and Ünsal, (2004); Uzun et al. (2004); Tuzlacı and Bulut, (2007); Ugurlu and Secmen, (2008); Tuzlaci et al. (2010); Tuzlacı and Ii̇, (2011); Bulut and Tuzlacı, (2015); Korkmaz and Si̇, (2015); Paksoy et al. (2016)
^b^ *Allium sphaerocephalum* L.	Amaryllidaceae				G16 (1)	Malamas and Marselos, (1992)
^a^ *Aloe vera* (L.) Burm.f.	Xanthorrhoeaceae		+	+	G2 (1), G4 (2), G8 (1), G9 (1), G16 (1), G17 (1), G28 (1), G30 (1), G32 (1), G33 (5)	Hanlidou et al. (2004); Yöney et al. (2010); Karousou and Deirmentzoglou, (2011); Adamidou, (2012); Lardos and Heinrich, (2013); Güler et al. (2015a); Güzel et al. (2015); Tsioutsiou et al. (2019)
^a^ *Aloysia citriodora* Palau	Verbenaceae			+	G4 (1), G12 (1), G22 (1)	Güzel et al. (2015); Petrakou et al. (2020)
^a^ *Althaea officinalis* L.	Malvaceae			+	G1 (2), G6 (4), G9 (1), G16 (1), G18 (1), G28 (1), G33 (1)	Sezik et al. (2001); Hanlidou et al. (2004); Everest and Ozturk, (2005); Ugulu et al. (2009); Adamidou, (2012); Güler et al. (2015a); Petrakou et al. (2020)
*Amaranthus retroflexus* L.	Amaranthaceae				G3 (1)	Axiotis et al. (2018)
^a^ *Anagallis arvensis* L. var. *caerulea* (L.) Gouan (= *Anagallis caerulea* L.)	Primulaceae			+	G4 (1), G33 (1)	Brussell, (2004); Güzel et al. (2015)
^a^ *Anethum graveolens* L.	Apiaceae			+	G2 (1), G3 (1), G4 (1), G16 (1), G22 (1)	Pieroni et al. (2005b); Akaydin et al. (2013); Sargin et al. (2013); Akgül et al. (2016); Petrakou et al. (2020)
^a^ *Apium graveolens* L.	Apiaceae			+	G5 (1), G29 (1), G33 (1)	Hanlidou et al. (2004); Everest and Ozturk, (2005); Güler et al. (2015a)
*Arbutus unedo* L.	Ericaceae			+	G4 (2), G22 (1)	Tuzlacı and Aymaz, (2001); Hanlidou et al. (2004); Güler et al. (2015a)
^a^ *Arctium lappa* L.	Asteraceae				G9 (1), G33 (1)	Tsioutsiou et al. (2019)
^a^ *Arctium minus* (Hill) Bernh.	Asteraceae			+	G1 (1), G2 (1), G4 (1), G5 (4), G9 (4), G16 (1), G22 (2), G33 (2)	Tabata et al. (1994); Fujita et al. (1995); Sezik et al. (1997); Tuzlacı and Erol, (1999); Yeşilada et al. (1999); Brussell, (2004); Kargioǧlu et al. (2008); Altundag and Ozturk, (2011); Kaval et al. (2014); Mükemre et al. (2015)
*Arnica montana* L.	Asteraceae				G10 (1), G32 (1), G33 (2)	Hanlidou et al. (2004); Petrakou et al. (2020)
^a^ *Artemisia absinthium* L.	Asteraceae	+	+	+	G2 (1), G4 (3), G5 (1), G8 (1), G9 (1), G16 (1), G17 (1), G22 (1), G33 (5), G37 (1)	Vokou et al. (1993); Karaman and Kocabas, (2001); Hanlidou et al. (2004); Uzun et al. (2004); Everest and Ozturk, (2005); Kültür, (2007); Karousou and Deirmentzoglou, (2011); Özüdoru et al. (2011); Pieroni et al. (2014b); Ahmet Sargin, (2015); Sargin et al. (2015a); Mükemre et al. (2015)
^b^ *Artemisia arborescens* (Vaill.) L.	Asteraceae				G4 (1), G33 (1)	Axiotis et al. (2018)
^b^ *Arum italicum* Mill.	Araceae	+		+	G9 (1), G16 (2), G17 (2), G22 (4), G33 (1)	Vokou et al. (1993); Yeşilada et al. (1999); Ecevit Genç and Özhatay, (2006); Tuzlaci and Alparslan, (2007); Pieroni, (2017); Gürbüz et al. (2019)
*Asphodeline lutea* (L.) Rchb.	Xanthorrhoeaceae			+	G2 (1), G22 (1), G29 (1), G34 (1)	Brussell, (2004); Kargioǧlu et al. (2008)
^a^ *Asphodelus aestivus* Brot.	Xanthorrhoeaceae		+	+	G8 (1), G17 (2), G22 (7), G27 (1), G30 (1), G32 (1), G33 (9)	Tuzlacı and Aymaz, (2001); Tuzlacı and Bulut, (2007); Tuzlacı and Sadıkoğlu, (2007); González-Tejero et al. (2008); Ugurlu and Secmen, (2008); Ugulu et al. (2009); Polat and Satıl, (2012); Uysal et al. (2012); Gürdal and Kültür, (2013); Bulut and Tuzlacı, (2015); Güzel et al. (2015); Bulut, (2016); Axiotis et al. (2018)
*Betula pendula* Roth	Betulaceae				G12 (1)	Hanlidou et al. (2004)
*Calendula arvensis* M.Bieb.	Asteraceae		+	+	G1 (1), G4 (2), G17 (1), G20 (2), G22 (1), G28 (2), G32 (3), G33 (3), G36 (1)	Brussell, (2004); González-Tejero et al. (2008); Sargin et al. (2013); Sargin et al. (2015a); Korkmaz et al. (2016a); Uzun and Kaya, (2016); Axiotis et al. (2018); Papageorgiou et al. (2020)
*Calendula officinalis* L.	Asteraceae		+	+	G1 (2), G2 (1), G4 (3), G10 (2), G16 (1), G17 (3), G20 (2), G27 (2), G31 (1), G33 (4)	Brussell, (2004); Hanlidou et al. (2004); Kültür, (2007); Ugulu et al. (2009); Yöney et al. (2010); Ünsal et al. (2010); Karousou and Deirmentzoglou, (2011); Yeşilyurt et al. (2017); Petrakou et al. (2020)
*Calendula* sp.	Asteraceae				G16 (1), G28 (1), G33 (1)	Adamidou, (2012)
^a^ *Calluna vulgaris* (L.) Hull	Ericaceae			+	G6 (1), G36 (1)	Ahmet Sargin, (2015); Petrakou et al. (2020)
*Camellia sinensis* (L.) Kuntze	Theaceae			+	G16 (1), G37 (1)	Hanlidou et al. (2004); Korkmaz et al. (2016a)
*Capsella bursa-pastoris* (L.) Medik.	Brassicaceae		+	+	G3 (9), G22 (1), G32 (1), G33 (4), G34 (3)	Brussell, (2004); Everest and Ozturk, (2005); González-Tejero et al. (2008); Ugulu et al. (2009); Cakilcioglu and Turkoglu, (2010); Ünsal et al. (2010); Altundag and Ozturk, (2011); Cakilcioglu et al. (2011); Bulut and Tuzlaci, (2013); Sargin et al. (2013); Charalampidou, (2014); Ahmet Sargin, (2015); Güler et al. (2015b); Güzel et al. (2015); Axiotis et al. (2018); Sargin and Büyükcengiz, (2019); Güler et al. (2020)
^a^ *Cardopatium corymbosum* (L.) Pers.	Asteraceae		+	+	G4 (2), G33 (2)	Pieroni et al. (2006); Tuzlacı and Bulut, (2007); Papageorgiou et al. (2020)
*Carthamus lanatus* L.	Asteraceae				G32 (1), G33 (1)	Axiotis et al. (2018)
^a^ *Centaurea cyanus* L. (synonym of *Cyanus segetum* Hill)	Asteraceae				G4 (1)	Axiotis et al. (2018)
^a^ *Centaurium erythraea* Rafn	Gentianaceae		+	+	G4 (1), G5 (1), G17 (2), G22 (2), G32 (1), G33 (4)	Tuzlaci and Tolon, (2000); Tuzlacı and Aymaz, (2001); Everest and Ozturk, (2005); Ecevit Genç and Özhatay, (2006); González-Tejero et al. (2008); Sargin et al. (2013); Karcı et al. (2017); Axiotis et al. (2018)
*Centella asiatica* (L.) Urb.	Apiaceae				G12 (2), G22 (1)	Hanlidou et al. (2004); Petrakou et al. (2020)
*Centranthus ruber* (L.) DC.	Caprifoliaceae				G2 (1)	Brussell, (2004)
*Cerastium glomeratum* Thuill.	Caryophyllaceae				G3 (1)	Axiotis et al. (2018)
*Ceratonia sliqua* L.	Leguminosae				G18 (1)	Brussell, (2004)
^a^ *Chelidonium majus* L.	Papaveraceae	+	+	+	G2 (1), G3 (1), G4 (4), G8 (1), G9 (1), G17 (2), G20 (7), G22 (1), G23 (2), G29 (1), G32 (1), G33 (2), G34 (1)	Vokou et al. (1993); Uzun et al. (2004); Kültür, (2007); Ünsal et al. (2010); Karousou and Deirmentzoglou, (2011); Sargin et al. (2013); Pieroni et al. (2014a); Ari et al. (2015); Sargin et al. (2015a); Polat et al. (2015); Gürbüz et al. (2019); Petrakou et al. (2020)
*Chenopodium album* L.	Amaranthaceae				G33 (1), G36 (1)	Axiotis et al. (2018)
^a^ *Cichorium intybus* L.	Asteraceae			+	G1 (1), G2 (1), G4 (3), G5 (1), G9 (1), G17 (3), G20 (1), G22 (6), G23 (1), G28 (1), G31 (2), G32 (1), G33 (8), G36 (1)	Sezik et al. (1991); Yeşilada et al. (1999); Sezik et al. (2001); Hanlidou et al. (2004); Everest and Ozturk, (2005); Tuzlaci et al. (2010); Çakilcioǧlu et al. (2010); Altundag and Ozturk, (2011); Adamidou, (2012); Özgen et al. (2012); Tetik et al. (2013); Sargin et al. (2015a); Güler et al. (2015b); Mükemre et al. (2015); Gürbüz et al. (2019); Karakaya et al. (2019); Karakaya et al. (2020); Güler et al. (2020)
^b^ *Cichorium spinosum* L.	Asteraceae				G32 (1), G36 (1)	Axiotis et al. (2018)
^a^ *Cistus creticus* L.	Cistaceae		+	+	G3 (2), G4 (1), G6 (1), G16 (1), G32 (1), G33 (3)	Honda et al. (1996); Tuzlacı and Aymaz, (2001); Pieroni et al. (2006); Polat and Satıl, (2012); Uysal et al. (2012); Akyol and Altan, (2013); Bulut and Tuzlacı, (2015); Kalankan et al. (2015); Axiotis et al. (2018)
^a^ *Cistus salviifolius* L.	Cistaceae			+	G3 (1), G5 (1), G16 (1), G33 (3), G36 (1)	Tuzlacı and Aymaz, (2001); Brussell, (2004); Kültür, (2007); Polat and Satıl, (2012); Bulut and Tuzlacı, (2015)
^a^ *Cistus* sp.	Cistaceae			+	G1 (1), G17 (1), G22 (1), G33 (1)	Sezik et al. (1991); Papageorgiou et al. (2020)
^a^ *Clematis vitalba* L.	Ranunculaceae	+			G2 (1), G4 (1), G34 (1)	Vokou et al. (1993); Pieroni, (2017)
^a^ *Cornus mas* L.	Cornaceae	+		+	G4 (3), G6 (1), G16 (1), G20 (1), G25 (1), G33 (1)	Brussell, (2004); Kültür, (2007); Tuzlaci et al. (2010); Uzun and Kaya, (2016); Pieroni and Sõukand, (2017)
^b^ *Crepis zacintha* (L.) Babc.	Asteraceae			+	G20 (1), G22 (1)	Kültür, (2007); Papageorgiou et al. (2020)
*Cupressus sempervirens* L.	Cupressaceae			+	G2 (1), G4 (2), G20 (2), G22 (2)	Polat and Satıl, (2012); Sargin et al. (2013); Sargin et al. (2015a); Bulut and Tuzlacı, (2015); Yeşilyurt et al. (2017); Axiotis et al. (2018)
*Curcuma longa* L.	Zingiberaceae				G4 (1)	Hanlidou et al. (2004)
*Cuscuta campestris* Yunck.	Convolvulaceae				G16 (1)	Tsioutsiou et al. (2019)
*Cuscuta* sp.	Convolvulaceae			+	G22 (1), G33 (1)	Akgül et al. (2016); Papageorgiou et al. (2020)
^a^ *Cydonia oblonga* Mill. (= *Cydonia vulgaris* Pers.)	Rosaceae	+		+	G1 (1), G4 (3), G6 (1), G22 (4), G23 (1), G28 (1), G33 (2), G37 (1)	Vokou et al. (1993); Tuzlacı and Aymaz, (2001); Everest and Ozturk, (2005); Ecevit Genç and Özhatay, (2006); Tuzlacı and Sadıkoğlu, (2007); Çakilcioǧlu and Türkoğlu, (2009); Pieroni et al. (2014b); Korkmaz et al. (2016a); Korkmaz et al. (2016b); Günbatan et al. (2016); Uzun and Kaya, (2016); Yeşilyurt et al. (2017)
^b^ *Cynoglossum creticum* Mill.	Boraginaceae			+	G4 (1), G9 (1), G20 (3), G33 (1), G36 (1)	Yeşilada et al. (1995); Sargin et al. (2013); Sargin et al. (2015a); Tsioutsiou et al. (2019)
*Datura stramonium* L.	Solanaceae			+	G1 (1), G4 (2), G9 (2), G10 (1), G14 (1), G17 (1), G22 (1), G33 (1)	Sezik et al. (1991); Sezik et al. (2001); Uzun et al. (2004); Ecevit Genç and Özhatay, (2006); Kültür, (2007); Tuzlacı and Sadıkoğlu, (2007); Axiotis et al. (2018)
^b^ *Delphinium staphisagria* L.	Ranunculaceae				G2 (1), G25 (1)	Hanlidou et al. (2004)
*Dioscorea balcanica* Kosanin.	Dioscoreaceae				G28 (1), G33 (1)	Brussell, (2004)
*Dittrichia graveolens* (L.) Greuter	Asteraceae				G25 (1)	Pieroni et al. (2006)
*Dittrichia viscosa* (L.) Greuter (= *Inula viscosa* (L.) Aiton)	Asteraceae		+	+	G3 (2), G4 (1), G9 (1), G32 (1), G33 (1)	González-Tejero et al. (2008); Güzel et al. (2015); Papageorgiou et al. (2020)
*Echinacea angustifolia* DC.	Asteraceae		+		G1 (1), G4 (2), G6 (1), G8 (1), G9 (2), G16 (3), G17 (1), G23 (1), G27 (1), G32 (1), G33 (1)	Hanlidou et al. (2004); Karousou and Deirmentzoglou, (2011); Petrakou et al. (2020)
^b^ *Elaeagnus rhamnoides* (L.) A.Nelson (= *Hippophaes rhamnoides* L.)	Elaeagnaceae				G1 (1), G13 (1), G17 (1), G27 (1), G28 (1), G33 (1), G37 (1)	Adamidou, (2012); Petrakou et al. (2020)
^a^ *Elettaria cardamomum* (L.) Maton	Zingiberaceae				G4 (1), G13 (2)	Hanlidou et al. (2004); Adamidou, (2012); Petrakou et al. (2020)
*Ephedra foeminea* Forssk.	Ephedraceae				G8 (1), G17 (1)	Hanlidou et al. (2004)
^a^ *Equisetum arvense* L.	Equisetaceae			+	G2 (2), G3 (3), G4 (1), G5 (1), G20 (2), G22 (1), G33 (1), G34 (1)	Vokou et al. (1993); Karaman and Kocabas, (2001); Everest and Ozturk, (2005); Toksoy et al. (2010); Özüdoru et al. (2011); Sargin et al. (2015a); Petrakou et al. (2020)
^a^ *Equisetum* sp.	Equisetaceae		+		G2 (1), G3 (2), G33 (1)	Hanlidou et al. (2004); Karousou and Deirmentzoglou, (2011)
*Erica arborea* L.	Ericaceae			+	G1 (1), G4 (1), G6 (1), G8 (1), G28 (1)	Brussell, (2004); Ecevit Genç and Özhatay, (2006); Tuzlacı and Bulut, (2007); Gürbüz et al. (2019)
*Erodium cicutarium* (L.) L'Hér	Geraniaceae			+	G3 (1), G16 (2), G20 (1), G22 (1), G34 (1)	Özgen et al. (2012); Güneş et al. (2017); Axiotis et al. (2018); Pieroni and Cattero, (2019)
*Eucalyptus camaldulensis* Dehnh.	Myrtaceae		+	+	G4 (2), G32 (1)	González-Tejero et al. (2008); Axiotis et al. (2018); Sargin and Büyükcengiz, (2019)
*Eucalyptus globulus* Labill.	Myrtaceae				G4 (2)	(Hanlidou et al., 2004; Petrakou et al., 2020)
^a^ *Euphorbia helioscopia* L.	Euphorbiaceae	+	+	+	G6 (1), G8 (1), G16 (1), G17 (1), G20 (2), G22 (1), G29 (1)	Brussell, (2004); Pieroni et al. (2005a); Yöney et al. (2010); Demirci and Özhatay, (2012); Güneş et al. (2017); Gürbüz et al. (2019)
^c^ *Euphorbia peplus* L.	Euphorbiaceae			+	G20 (1), G34 (1)	Brussell, (2004); Demirci and Özhatay, (2012)
*Euphrasia salisburgensis* Funck ex Hoppe	Orobanchaceae				G4 (2)	Hanlidou et al. (2004); Petrakou et al. (2020)
^b^ *Ferula communis* L.	Apiaceae				G33 (1)	Brussell, (2004)
^a^ *Ficaria verna* Huds. (= *Ficaria ranunculoides* Roth. = *Ranunculus ficaria* L.)	Ranunculaceae			+	G5 (1), G9 (1), G20 (1), G22 (4), G32 (1), G33 (2)	Tuzlacı and Aymaz, (2001); Brussell, (2004); Ugurlu and Secmen, (2008); Sarper et al. (2009); Ahmet Sargin, (2015); Ari et al. (2015); Güzel et al. (2015); Yeşilyurt et al. (2017)
^a^ *Ficus carica* L.	Moraceae	+	+	+	G3 (2), G4 (1), G5 (1), G8 (1), G9 (5), G11 (2), G16 (9), G17 (6), G20 (28), G22 (13), G32 (2), G33 (2)	Axiotis et al. (2018), Ugulu et al. (2009), Altundag and Ozturk, (2011), Karakaya et al. (2019), Hanlidou et al. (2004), Sezik et al. (1997), Çakilcioǧlu and Türkoğlu, (2009), Güzel et al. (2015), Pieroni et al. (2014a), Gürbüz et al. (2019), Papageorgiou et al. (2020), Malamas and Marselos, (1992), Fujita et al. (1995); Yeşilada et al. (1995); Yeşilada et al. (1999); Yöney et al. (2010); Lardos and Heinrich, (2013), Kargioǧlu et al. (2008), Polat et al. (2015), Uzun and Kaya, (2016), Karcı et al. (2017), Karaman and Kocabas, (2001), Tuzlacı and Bulut, (2007); Ugurlu and Secmen, (2008); Tuzlaci et al. (2010), Bulut and Tuzlacı, (2015), Güler et al. (2015a), Sargin et al. (2013), Tuzlacı and Aymaz, (2001), Tuzlacı and Erol, (1999), Sargin et al. (2015a), Ahmet Sargin, (2015), Ecevit Genç and Özhatay, (2006), Bulut, (2016), González-Tejero et al. (2008), Polat and Satıl, (2012), Tuzlaci and Tolon, (2000); Bulut and Tuzlaci, (2013); Güler et al. (2015b); Sargin and Büyükcengiz, (2019); Güler et al. (2020), Honda et al. (1996), Akyol and Altan, (2013), Tuzlacı and Doğan, (2010); Sargin et al. (2015b); Bulut et al. (2017a)
*Filipendula hexapetala* Gilib. (synonym of *Filipendula vulgaris* Moench)	Rosaceae				G22 (1)	Brussell, (2004)
^a^ *Foeniculum vulgare* Mill.	Apiaceae			+	G4 (3), G5 (1)	Everest and Ozturk, (2005); Polat and Satıl, (2012); Petrakou et al. (2020)
*Fraxinus ornus* L.	Oleaceae			+	G20 (1), G22 (1), G34 (1)	Vokou et al. (1993); Kültür, (2007); Tuzlaci et al. (2010)
^a^ *Fumaria officinalis* L.	Papaveraceae			+	G6 (2), G8 (1), G17 (5), G22 (1), G27 (3), G30 (1)	Brussell, (2004); Tuzlacı and Sadıkoğlu, (2007); Ugulu et al. (2009); Altundag and Ozturk, (2011); Ahmet Sargin, (2015); Akgül et al. (2016); Korkmaz et al. (2016a); Güneş et al. (2017)
^a^ *Galium aparine* L.	Rubiaceae		+	+	G1 (1), G6 (1), G14 (1), G17 (1), G23 (1), G27 (1), G29 (1), G32 (1)	Hanlidou et al. (2004); González-Tejero et al. (2008); Güneş et al. (2017); Petrakou et al. (2020)
*Geranium asphodeloides* Burm.f.	Geraniaceae			+	G4 (2), G33 (1)	Brussell, (2004); Uzun et al. (2004)
*Geranium versicolor* L.	Geraniaceae				G20 (1)	Brussell, (2004)
^a^ *Glycyrrhiza glabra* L.	Leguminosae		+	+	G1 (1), G4 (2), G5 (2), G6 (1), G22 (2), G32 (2), G36 (1)	Honda et al. (1996); Sezik et al. (2001); Everest and Ozturk, (2005); Kargıoğlu et al. (2010); Toksoy et al. (2010); Karousou and Deirmentzoglou, (2011); Güzel et al. (2015); Karakaya et al. (2019); Petrakou et al. (2020)
*Hamamelis virginiana* L.	Hamamelidaceae		+		G3 (1), G4 (1), G22 (1), G33 (2)	Hanlidou et al. (2004); Yöney et al. (2010); Tsioutsiou et al. (2017)
*Harpagophytum procumbens* (Burch.) DC. ex Meisn.	Pedalidaceae				G21 (1), G32 (1)	Hanlidou et al. (2004); Petrakou et al. (2020)
^a^ *Hedera helix* L.	Araliaceae			+	G9 (3), G12 (2), G15 (1), G28 (1), G33 (2)	Vokou et al. (1993); Tuzlaci and Tolon, (2000); Brussell, (2004); Hanlidou et al. (2004); Ünsal et al. (2010); Yeşilyurt et al. (2017); Gürbüz et al. (2019); Petrakou et al. (2020)
^c^ *Helichrysum stoechas* (L.) Moench	Asteraceae			+	G3 (1), G4 (1), G5 (1), G17 (1), G33 (1)	Everest and Ozturk, (2005); Axiotis et al. (2018)
^b^ *Hypericum olympicum* L.	Hypericaceae			+	G9 (1), G22 (1), G33 (3)	Tuzlacı and Aymaz, (2001); Kalankan et al. (2015); Papageorgiou et al. (2020)
^a^ *Hypericum perforatum* L.	Hypericaceae	+	+	+	G3 (4), G4 (9), G5 (2), G6 (2), G7 (1), G9 (3), G17 (1), G20 (1), G22 (6), G23 (2), G28 (2), G31 (1), G32 (1), G33 (45), G34 (1)	(Vokou et al. (1993); Axiotis et al. (2018); Tsioutsiou et al. (2019); Brussell, (2004); Hanlidou et al. (2004); Tsioutsiou et al. (2017); Pieroni, (2017); Karousou and Deirmentzoglou, (2011); Güzel et al. (2015); Pieroni et al. (2014a); Everest and Ozturk, (2005); Sezik et al. (2001); Malamas and Marselos, (1992); Kültür, (2007); Polat et al. (2015); Uzun and Kaya, (2016); Güneş et al. (2017); Karaman and Kocabas, (2001); Tuzlacı and Bulut, (2007); Tuzlaci et al. (2010); Tuzlacı and Ii̇, (2011); Bulut and Tuzlacı, (2015); Korkmaz and Si̇, (2015); Akaydin et al. (2013); Sargin et al. (2013); Tuzlacı and Aymaz, (2001); Sargin et al. (2015a); Ahmet Sargin, (2015); Özüdoru et al. (2011); Ecevit Genç and Özhatay, (2006); Tuzlaci and Alparslan, (2007); Uysal et al. (2012); González-Tejero et al. (2008); Polat and Satıl, (2012); Korkmaz et al. (2016a); Charalampidou, (2014); Bulut and Tuzlaci, (2013); Sargin and Büyükcengiz, (2019); Tuzlaci and Tolon, (2000); Kalankan et al. (2015)
^a^ *Hypericum* sp.	Hypericaceae			+	G4 (2), G5 (1), G10 (2), G11 (1), G16 (1), G17 (1), G22 (2), G28 (1), G32 (1), G33 (4)	Pieroni et al. (2005b); Adamidou, (2012); Papageorgiou et al. (2020); Petrakou et al. (2020)
^b^ *Hypericum triquetrifolium* Turra	Hypericaceae		+	+	G4 (2), G22 (1), G27 (1), G32 (1), G33 (6)	Tuzlacı and Sadıkoğlu, (2007); González-Tejero et al. (2008); Tetik et al. (2013); Ahmet Sargin, (2015); Sargin et al. (2015b); Sargin and Büyükcengiz, (2019); Papageorgiou et al. (2020)
^a^ *Hyssopus officinalis* subsp. *aristatus* (Godr.) Nyman.	Lamiaceae				G34 (1)	Vokou et al. (1993)
^a^ *Juglans regia* L.	Juglandaceae	+	+	+	G1 (3), G2 (7), G3 (4), G4 (18), G5 (2), G6 (3), G8 (4), G9 (3), G11 (1), G14 (1), G16 (1), G17 (11), G19 (1), G20 (2), G22 (16), G23 (2), G27 (5), G28 (1), G29 (3), G30 (1), G32 (3), G33 (10), G34 (3), G36 (1), G37 (1)	Nadiroğlu et al. (2019); Ugulu et al. (2009); Altundag and Ozturk, (2011); Karakaya et al. (2019); Karakaya et al. (2020); Hanlidou et al. (2004); Tsioutsiou et al. (2017); Pieroni and Sõukand, (2017); Sezik et al. (1997); Çakilcioǧlu and Türkoğlu, (2009); Güzel et al. (2015); Everest and Ozturk, (2005); Tetik et al. (2013); Gürbüz et al. (2019); Papageorgiou et al. (2020); Ari et al. (2015); Sezik et al. (2001); Malamas and Marselos, (1992); Yöney et al. (2010); Yeşilada et al. (1995); Yeşilada et al. (1999); Kültür, (2007); Kargioǧlu et al. (2008); Yildirim et al. (2008); Karcı et al. (2017); Karaman and Kocabas, (2001); Tuzlaci et al. (2010); Tuzlacı and Ii̇, (2011); Bulut and Tuzlacı, (2015); Paksoy et al. (2016); Güler et al. (2015a); Tabata et al. (1994); Kaval et al. (2014); Ahmet Sargin, (2015); Ecevit Genç and Özhatay, (2006); Tuzlacı and Sadıkoğlu, (2007); Polat and Satıl, (2012); Ünsal et al. (2010); Bulut and Tuzlaci, (2013); Sargin and Büyükcengiz, (2019); Tuzlaci and Tolon, (2000); Honda et al. (1996)
^a^ *Juniperus communis* L. (contains *Juniperus communis* L. subsp. *alpina* (Suter) Celak. = *Juniperus communis* L. subsp. *nana* (synonym of *Juniperus communis* var. *saxatilis* Pall.))	Cupressaceae	+		+	G4 (1), G17 (2), G22 (2), G28 (1), G30 (1), G32 (2), G33 (2)	Fujita et al. (1995); Özgen et al. (2012); Pieroni and Sõukand, (2017); Karakaya et al. (2019); Karakaya et al. (2020); Petrakou et al. (2020)
^b^ *Juniperus oxycedrus* L.	Cupressaceae			+	G2 (1), G4 (4), G9 (3), G17 (5), G22 (13), G27 (2), G28 (1), G29 (1), G30 (1), G32 (2), G33 (6), G34 (1)	Sezik et al. (1992); Yeşilada et al. (1993); Fujita et al. (1995); Yeşilada et al. (1995); Honda et al. (1996); Yeşilada et al. (1999); Karaman and Kocabas, (2001); Sezik et al. (2001); Ecevit Genç and Özhatay, (2006); Kültür, (2007); Ugurlu and Secmen, (2008); Ugulu et al. (2009); Çakilcioǧlu and Türkoğlu, (2009); Kargıoğlu et al. (2010); Demirci and Özhatay, (2012); Polat and Satıl, (2012); Bulut and Tuzlaci, (2013); Sargin et al. (2015a); Bulut and Tuzlacı, (2015); Özdemir and Alpınar, (2015); Günbatan et al. (2016); Bulut et al. (2017a); Yeşilyurt et al. (2017); Axiotis et al. (2018)
^b^ *Lamium garganicum* L.	Lamiaceae				G33 (1)	Brussell, (2004)
^a^ *Laurus nobilis* L.	Lauraceae		+	+	G1 (1), G2 (6), G3 (3), G4 (5), G5 (1), G6 (1), G8 (1), G14 (2), G16 (2), G17 (5), G18 (1), G22 (6), G27 (1), G28 (1), G30 (1), G32(6), G33 (3), G35 (1)	Honda et al. (1996); Tuzlacı and Erol, (1999); Tuzlaci and Tolon, (2000); Hanlidou et al. (2004); Pieroni et al. (2005b); Everest and Ozturk, (2005); González-Tejero et al. (2008); Ugurlu and Secmen, (2008); Ugulu et al. (2009); Toksoy et al. (2010); Karousou and Deirmentzoglou, (2011); Akaydin et al. (2013); Gürdal and Kültür, (2013); Charalampidou, (2014); Ahmet Sargin, (2015); Güzel et al. (2015); Polat et al. (2015); Akgül et al. (2016); Korkmaz et al. (2016a); Karcı et al. (2017); Axiotis et al. (2018); Gürbüz et al. (2019); Sargin and Büyükcengiz, (2019); Petrakou et al. (2020)
*Lavandula angustifolia* Mill.	Lamiaceae		+		G1 (1), G2 (1), G4 (3), G17 (1), G25 (1), G27 (1)	Hanlidou et al. (2004); Karousou and Deirmentzoglou, (2011); Petrakou et al. (2020)
*Lavandula stoechas* L.	Lamiaceae			+	G4 (3), G9 (1), G17 (2), G33 (1)	Uzun et al. (2004); Sekeroglu et al. (2006); Toksoy et al. (2010); Güler et al. (2015a); Axiotis et al. (2018)
^a^ *Lilium candidum* L.	Liliaceae			+	G33 (3), G36 (1)	Ugurlu and Secmen, (2008); Ugulu et al. (2009); Axiotis et al. (2018)
*Linaria elatine* (L.) Mill. (synonym of *Kickxia elatine* (L.) Dumort.)	Plantaginaceae				G2 (1)	Hanlidou et al. (2004)
^a^ *Linum usitatissimum* L.	Linaceae			+	G1 (2), G5 (1), G9 (6), G10 (1), G17 (1), G22 (2), G23 (1), G27 (1), G31 (1), G32 (1), G33 (2)	Fujita et al. (1995); Yeşilada et al. (1995); Yeşilada et al. (1999); Sezik et al. (2001); Hanlidou et al. (2004); Ugulu et al. (2009); Karakaya et al. (2020); Adamidou, (2012); Güler et al. (2015a); Tsioutsiou et al. (2019); Petrakou et al. (2020)
*Lycopersicon esculentum* Mill.	Solanaceae			+	G9 (4), G16 (1), G33 (1), G36 (1)	Malamas and Marselos, (1992); Fujita et al. (1995); Yeşilada et al. (1995); Sezik et al. (2001); Günbatan et al. (2016); Karcı et al. (2017)
*Lythrum salicaria* L.	Lythraceae				G33 (1)	Brussell, (2004)
^a^ *Malus domestica* Borkh.	Rosaceae			+	G23 (1), G32 (1), G33 (1)	Brussell, (2004); Güler et al. (2015a); Korkmaz et al. (2016b)
^a^ *Malva sylvestris* L.	Malvaceae	+	+	+	G1 (5), G2 (1), G4 (3), G5 (8), G6 (3), G8 (1), G9 (9), G10 (1), G11 (2), G16 (1), G17 (4), G22 (5), G28 (1), G31 (1), G32 (2), G33 (6), G34 (1), G36 (5)	Tuzlacı and Erol, (1999); Tuzlaci and Tolon, (2000); Gözüm and Ünsal, (2004); Hanlidou et al. (2004); Simsek et al. (2004); Everest and Ozturk, (2005); Ecevit Genç and Özhatay, (2006); Kültür, (2007); Tuzlacı and Bulut, (2007); González-Tejero et al. (2008); Ugulu et al. (2009); Kargıoğlu et al. (2010); Tuzlaci et al. (2010); Yöney et al. (2010); Altundag and Ozturk, (2011); Karakaya et al.(2020); Adamidou, (2012); Polat and Satıl, (2012); Akaydin et al. (2013))); Polat et al. (2013); Sargin et al. (2013); Pieroni et al. (2014a); Sargin et al. (2015a); Sargin et al. (2015b); Güzel et al. (2015); Korkmaz et al. (2016a); Axiotis et al. (2018); Akbulut et al. (2019); Polat, (2019); Güler et al. (2020); Petrakou et al. (2020)
^a^ *Matricaria chamomilla* L. (= *Matricaria recutita* L.)	Asteraceae		+	+	G1 (3), G2 (2), G4 (5), G5 (5), G6 (2), G8 (1), G9 (1), G14 (1), G17 (1), G22 (2), G25 (1), G28 (2), G32 (3), G33 (5)	Tuzlaci and Tolon, (2000); Gözüm and Ünsal, (2004); Hanlidou et al. (2004); Everest and Ozturk, (2005); Pieroni et al. (2006); Kültür, (2007); Tuzlaci et al. (2010); Karousou and Deirmentzoglou, (2011); Adamidou, (2012); Lardos and Heinrich, (2013); Bulut and Tuzlacı, (2015); Tsioutsiou et al. (2017); Axiotis et al. (2018); Sargin and Büyükcengiz, (2019); Tsioutsiou et al. (2019); Papageorgiou et al. (2020); Petrakou et al. (2020)
^b^ *Medicago sativa* L.	Leguminosae			+	G3 (4), G9 (1), G33 (2), G34 (1)	Altundag and Ozturk, (2011); Kaval et al. (2014); Sargin et al. (2015a); Axiotis et al. (2018)
^a^ *Melissa officinalis* L.	Lamiaceae		+	+	G1 (1), G4 (6), G16 (2), G33 (1), G37 (1)	Karaman and Kocabas, (2001); Uzun et al. (2004); Everest and Ozturk, (2005); Toksoy et al. (2010); Tuzlacı and Doğan, (2010); Altundag and Ozturk, (2011); Karousou and Deirmentzoglou, (2011); Adamidou, (2012); Petrakou et al. (2020)
^a^ *Mentha spicata* L.	Lamiaceae			+	G4 (1), G5 (1), G6 (1), G22 (1), G33 (1)	Tuzlacı and Aymaz, (2001); Pieroni et al. (2005b); Axiotis et al. (2018); Petrakou et al. (2020)
^a^ *Mentha* sp.	Lamiaceae			+	G22 (1), G33 (1)	Adamidou, (2012); Akaydin et al. (2013)
^a^ *Mentha × piperita* L.	Lamiaceae			+	G4 (1), G17 (1)	Gürbüz et al. (2019); Petrakou et al. (2020)
*Micromeria juliana* (L.) Benth. ex Rchb.	Lamiaceae				G4 (1), G20 (1)	Axiotis et al. (2018)
*Momordica charantia* L.	Cucurbitaceae			+	G5 (1), G9 (1), G11 (1), G17 (2), G22 (3), G27 (2), G31 (1), G33 (10)	Yeşilada et al. (1999); Uzun et al. (2004); Kültür, (2007); Ugulu et al. (2009); Tuzlaci et al. (2010); Polat and Satıl, (2012); Akaydin et al. (2013); Ahmet Sargin, (2015); Sargin et al. (2015b); Güzel et al. (2015); Tsioutsiou et al. (2017); Sargin and Büyükcengiz, (2019); Tsioutsiou et al. (2019); Güler et al. (2020)
^b^ *Morus alba* L.	Moraceae			+	G3 (1), G4 (1), G5 (1), G6 (3), G9 (4), G17 (2), G23 (1), G33 (1)	Axiotis et al. (2018); Altundag and Ozturk, (2011); Sezik et al. (1997); Gürbüz et al. (2019); Güneş et al. (2017); Tuzlacı and Bulut, (2007); Tuzlaci et al. (2010); Tuzlacı and Ii̇, (2011); Bulut and Tuzlacı, (2015); Güler et al. (2015a); Akaydin et al. (2013); Ecevit Genç and Özhatay, (2006)
^a^ *Myrtus communis* L.	Myrtaceae		+	+	G1 (1), G2 (3), G3 (3), G4 (5), G5 (1), G6 (1), G9 (3), G17 (1), G22 (2), G28 (4), G30 (1), G32 (2), G33 (4)	Yeşilada et al. (1995); Tuzlacı and Erol, (1999); Everest and Ozturk, (2005; González-Tejero et al. (2008); Ugulu et al. (2009); Yöney et al. (2010); Karousou and Deirmentzoglou, (2011); Uysal et al. (2012); Bulut and Tuzlaci, (2013); Ahmet Sargin, (2015); Sargin et al. (2015a); Güzel et al. (2015); Bulut et al. (2017a); Axiotis et al. (2018); Akbulut et al. (2019)
^a^ *Nasturtium officinale* R.Br.	Brassicaceae			+	G4 (1), G5 (1), G13 (1), G17 (1)	Hanlidou et al. (2004); Sargin et al. (2013); Polat, (2019)
^a^ *Nerium oleander* L.	Apocynaceae		+	+	G4 (1), G8 (1), G9 (1), G16 (3), G17 (2), G22 (1), G25 (2), G30 (2), G33 (1), G34 (1), G36 (1)	Yeşilada et al. (1995); Tuzlacı and Erol, (1999); Brussell, (2004); Tuzlacı and Sadıkoğlu, (2007); Yöney et al. (2010); Akyol and Altan, (2013); Gürdal and Kültür, (2013); Ahmet Sargin, (2015); Sargin et al. (2015a); Güzel et al. (2015); Güneş et al. (2017); Karcı et al. (2017); Akbulut et al. (2019)
*Nicotiana tabacum* L.	Solanaceae	+		+	G3 (7), G4 (3), G5 (1), G25 (1), G33 (5)	Tabata et al. (1994); Pieroni et al. (2005b); Kültür, (2007); Pieroni et al. (2014a); Pieroni et al. (2014c); Karcı et al. (2017); Pieroni, (2017); Pieroni and Sõukand, (2017); Gürbüz et al. (2019); Tsioutsiou et al. (2019)
*Ocimum basilicum* L.	Lamiaceae		+	+	G5 (2), G6 (2), G9 (1), G16 (3), G22 (1), G33 (1)	Sezik et al. (1992); Everest and Ozturk, (2005); Karousou and Deirmentzoglou, (2011); Sargin et al. (2013); Güler et al. (2015a); Akgül et al. (2016); Bulut et al. (2019); Petrakou et al. (2020)
^a^ *Olea europaea* L.	Oleaceae		+	+	G1 (2), G2 (1), G4 (3), G5 (1), G6 (4), G9 (2), G10 (2), G16 (1), G20 (2), G22 (2), G31 (1), G32 (3), G33 (12), G36 (2)	Fujita et al. (1995); Honda et al. (1996); Sezik et al. (1997); Yeşilada et al. (1999); Tuzlacı and Aymaz, (2001); Pieroni et al. (2005b); Everest and Ozturk, (2005); Pieroni et al. (2006); Kültür, (2007); Tuzlacı and Bulut, (2007); Karousou and Deirmentzoglou, (2011); Akaydin et al. (2013); Bulut and Tuzlaci, (2013); Gürdal and Kültür, (2013); Lardos and Heinrich, (2013); Ahmet Sargin, (2015); Bulut and Tuzlacı, (2015); Güzel et al. (2015); Günbatan et al. (2016); Karcı et al. (2017); Axiotis et al. (2018); Gürbüz et al. (2019); Karakaya et al. (2019); Sargin and Büyükcengiz, (2019)
^a^ *Origanum dictamnus* L.	Lamiaceae		+		G1 (1), G3 (1), G4 (3), G9 (1), G10 (2), G20 (1), G32 (1), G33 (4)	Hanlidou et al. (2004); Karousou and Deirmentzoglou, (2011); Adamidou, (2012); Tsioutsiou et al. (2017); Petrakou et al. (2020)
^a^ *Origanum majorana* L. (= *Origanum dubium* Boiss.)	Lamiaceae		+	+	G4 (3), G5 (1)	Brussell, (2004); Everest and Ozturk, (2005); Karousou and Deirmentzoglou, (2011)
^a^ *Origanum vulgare* L.	Lamiaceae			+	G1 (1), G2 (1), G4 (3), G6 (2), G22 (2), G33 (3)	Tabata et al. (1994); Tuzlacı and Aymaz, (2001); Hanlidou et al. (2004); Özgökçe and Özçelik, (2004); Çakilcioǧlu and Türkoğlu, (2009); Altundag and Ozturk, (2011); Sargin et al. (2015a); Papageorgiou et al. (2020); Petrakou et al. (2020)
*Paeonia mascula* (L.) Mill.	Paeoniaceae			+	G3 (1), G21 (1), G22 (1)	Ugulu et al. (2009); Axiotis et al., 2018)
^b^ *Parietaria judaica* L.	Urticaceae		+	+	G3 (1), G6 (1), G17 (2), G32 (1), G33 (1)	Sezik et al. (2001); González-Tejero et al. (2008); Ugulu et al. (2009); Güzel et al. (2015); Axiotis et al. (2018)
^a^ *Parietaria officinalis* L.	Urticaceae				G10 (1), G33 (1)	Brussell, (2004)
^a^ *Pimpinella anisum* L.	Apiaceae			+	G4 (2), G33 (1)	Hanlidou et al. (2004); Everest and Ozturk, (2005)
^b^ *Pinus brutia* Ten.	Pinaceae		+	+	G1 (1), G3 (1), G4 (1), G5 (1), G9 (2), G22 (1), G27 (1), G28 (1), G30 (1), G32 (1), G33 (6)	Honda et al. (1996); Sezik et al. (2001); González-Tejero et al. (2008; Kargıoğlu et al. (2010); Demirci and Özhatay, (2012); Polat and Satıl, (2012); Ahmet Sargin, (2015); Bulut and Tuzlacı, (2015); Bulut et al. (2017a); Güneş et al. (2017); Axiotis et al. (2018)
^a^ *Pistacia lentiscus* L.	Anacardiaceae		+	+	G4 (1), G6 (2), G33 (2)	Pieroni et al. (2005b); Pieroni et al. (2006); Ahmet Sargin, (2015); Axiotis et al. (2018)
^b^ *Plantago lanceolata* L.	Plantaginaceae	+	+	+	G1 (2), G3 (3), G4 (1), G5 (4), G9 (20), G16 (4), G17 (1), G22 (6), G25 (1), G28 (1), G32 (1), G33 (31), G34 (3), G36 (2)	Şanda and Küçüködük (2018); Nadiroğlu et al. (2019); Vokou et al. (1993); Brussell, (2004); Ugulu et al. (2009); Altundag and Ozturk, (2011); Karakaya et al. (2020); Tsioutsiou et al. (2017); Pieroni and Sõukand, (2017); Pieroni et al. (2014a); Tetik et al. (2013); Gürbüz et al. (2019); Sezik et al. (2001); Malamas and Marselos, (1992); Fujita et al. (1995); Yeşilada et al. (1995); Kültür, (2007); Hayta et al. (2014); Polat et al. (2015); Uzun and Kaya, (2016); Güneş et al. (2017); Karcı et al. (2017); Tuzlacı and Ii̇, (2011); Tuzlacı and Aymaz, (2001); Tabata et al. (1994); Kaval et al. (2014); Mükemre et al. (2015); Ecevit Genç and Özhatay, (2006); González-Tejero et al. (2008); Polat and Satıl, (2012); Charalampidou, (2014); Bulut and Tuzlaci, (2013); Tuzlaci and Tolon, (2000); Kalankan et al. (2015); Bulut et al. (2017a); Kargıoğlu et al. (2010); Cakilcioglu et al. (2007); Polat et al. (2013); Özdemir and Alpınar, (2015); Ertuǧ, (2000); Özgökçe and Özçelik, (2004); Polat, (2019)
^a^ *Plantago major* L.	Plantaginaceae	+		+	G1 (3), G3 (7), G4 (3), G5 (6), G8 (4), G9 (44), G11 (1), G16 (3), G17 (4), G19 (1), G20 (4), G22 (10), G23 (1), G28 (3), G33 (43), G36 (3)	Nadiroğlu et al. (2019); Altundag and Ozturk, (2011); Karakaya et al. (2019); Karakaya et al. (2020); Hanlidou et al. (2004); Tsioutsiou et al. (2017); Pieroni and Sõukand, (2017); Sezik et al. (1997); Adamidou, (2012); Pieroni et al. (2014a); Pieroni, (2008); Everest and Ozturk, (2005); Tetik et al. (2013); Gürbüz et al. (2019); Sezik et al. (2001); Pieroni et al. (2005a); Fujita et al. (1995); Yeşilada et al. (1995); Yeşilada et al. (1999); Pieroni et al. (2005b); Ezer and Mumcu Arisan, (2006); Kültür, (2007); Kargioǧlu et al. (2008); Hayta et al. (2014); Polat et al. (2015); Günbatan et al. (2016); Karcı et al. (2017); Karaman and Kocabas, (2001); Uzun et al. (2004); Tuzlacı and Bulut, (2007); Tuzlacı and Ii̇, (2011); Bulut and Tuzlacı, (2015); Korkmaz and Si̇, (2015); Güler et al. (2015a); Sargin et al. (2013); Tuzlacı and Aymaz, (2001); Tabata et al. (1994); Kaval et al. (2014); Mükemre et al. (2015); Sargin et al. (2015a); Ahmet Sargin, (2015); Özüdoru et al. (2011)
^a^ *Plantago media* L.	Plantaginaceae			+	G5 (2), G9 (3), G22 (2), G32 (1), G33 (1), G34 (1)	(Altundag and Ozturk, 2011; Mükemre et al., 2015; Korkmaz et al., 2016a; Dalar et al., 2018; Karakaya et al., 2019; Tsioutsiou et al., 2019)
^a^ *Platanus orientalis* L.	Platanaceae			+	G3 (2), G4 (1), G5 (1), G8 (1), G9 (3), G22 (2), G33 (5), G36 (1)	Yeşilada et al. (1995); Karaman and Kocabas, (2001); Hanlidou et al. (2004); Tuzlacı and Sadıkoğlu, (2007); Ugulu et al. (2009); Polat and Satıl, (2012); Bulut and Tuzlaci, (2013); Sargin et al. (2013); Sargin et al. (2015a); Uzun and Kaya, (2016); Petrakou et al. (2020)
*Plumbago europaea* L.	Plumbaginaceae			+	G2 (4), G4 (2), G5 (1), G8 (1), G17 (4), G26 (1), G30 (1), G33 (2), G36 (1)	Fujita et al. (1995); Sezik et al. (2001); Brussell, (2004); Tuzlacı and Bulut, (2007); Ugurlu and Secmen, (2008); Kargıoğlu et al. (2010); Bulut and Tuzlacı, (2015); Güzel et al. (2015); Bulut et al. (2017b); Axiotis et al. (2018)
^a^ *Polygonatum multiflorum* (L.) All.	Asparagaceae				G10 (1)	Brussell, (2004)
^a^ *Polygonum aviculare* L.	Polygonaceae		+		G3 (2), G32 (1), G33 (1)	Hanlidou et al. (2004); González-Tejero et al. (2008); Axiotis et al. (2018)
*Populus alba* L.	Salicaceae			+	G3 (1), G4 (1), G14 (1), G32 (1), G33 (1)	Sargin et al. (2013); Axiotis et al. (2018); Karakaya et al. 2020
*Populus tremula* L.	Salicaceae			+	G6 (1), G20 (1)	Kültür, (2007); Charalampidou, (2014)
^b^ *Potentilla recta* L.	Rosaceae			+	G3 (1), G5 (1), G33 (1)	Özdemir and Alpınar, (2015); Axiotis et al. (2018)
*Primula veris* L.	Primulaceae	+			G3 (1), G4 (1)	Hanlidou et al. (2004); Pieroni et al. (2014c)
^a^ *Pteridium aquilinum* (L.) Kuhn	Dennstaedtiaceae			+	G4 (1), G17 (1)	Hanlidou et al. (2004); Bulut and Tuzlacı, (2015)
*Pyrus amygdaliformis* Vill.	Rosaceae			+	G4 (1), G16 (3)	Brussell, (2004); Tuzlacı and Sadıkoğlu, (2007); Bulut and Tuzlacı, (2015); Güler et al. (2020)
^a^ *Quercus ilex* L.	Fagaceae				G22 (1)	Axiotis et al. (2018)
^a^ *Rhus coriaria* L.	Anacardiaceae			+	G3 (1), G4 (7), G6 (3), G9 (1), G16 (1), G17 (2), G20 (1), G22 (2), G32 (1), G33 (7), G36 (1)	Sezik et al. (1991); Yeşilada et al. (1993); Tabata et al. (1994); Yeşilada et al. (1995); Tuzlacı and Erol, (1999); Karaman and Kocabas, (2001); Everest and Ozturk, (2005); Cakilcioglu and Turkoglu, (2010); Çakilcioǧlu et al. (2010); Ünsal et al. (2010); Altundag and Ozturk, (2011); Demirci and Özhatay, (2012); Bulut and Tuzlaci, (2013); Hayta et al. (2014); Ahmet Sargin, (2015); Sargin et al. (2015b); Paksoy et al. (2016); Güneş et al. (2017); Axiotis et al. (2018)
^c^ *Rosa canina* L.	Rosaceae	+		+	G3 (1), G4 (3), G5 (3), G8 (4), G10 (1), G17 (7), G18 (1), G20 (1), G22 (31), G27 (1), G28 (2), G33 (5), G34 (1), G37 (1)	Tabata et al. (1994); Fujita et al. (1995); Yeşilada et al. (1995); Honda et al. (1996); Sezik et al. (1997); Tuzlacı and Erol, (1999); Yeşilada et al. (1999); Tuzlaci and Tolon, (2000); Sezik et al. (2001); Tuzlacı and Aymaz, (2001); Özgökçe and Özçelik, (2004); Pieroni et al. (2005a); Everest and Ozturk, (2005); Ecevit Genç and Özhatay, (2006); Ezer and Mumcu Arisan, (2006); Kültür, (2007); Tuzlaci and Alparslan, (2007); Ugurlu and Secmen, (2008); Çakilcioǧlu and Türkoğlu, (2009); Cakilcioglu and Turkoglu, (2010); Altundag and Ozturk, (2011); Bulut and Tuzlaci, (2013); Polat et al. (2013); Tetik et al. (2013); Pieroni et al. (2014a); Ahmet Sargin, (2015); Ari et al. (2015); Sargin et al. (2015a); Güzel et al. (2015); Han and Bulut, (2015); Uzun and Kaya, (2016); Yeşilyurt et al. (2017); Axiotis et al. (2018); Gürbüz et al. (2019); Karakaya et al. (2019); Karaköse et al. (2019); Polat, (2019); Papageorgiou et al. (2020)
^a^ *Rosa* sp.	Rosaceae	+		+	G4 (1), G6 (3), G32 (1), G33 (2)	Hanlidou et al. (2004); Pieroni et al. (2005b); Sargin et al. (2013); Pieroni, (2017); Yeşilyurt et al. (2017); Petrakou et al. (2020)
*Rosmarinus officinalis* L.	Lamiaceae		+	+	G2 (3), G4 (4), G5 (2), G7 (1), G14 (1), G32 (2), G33 (3), G36 (1), G37 (1)	Hanlidou et al. (2004); Everest and Ozturk, (2005); Tuzlacı and Sadıkoğlu, (2007); González-Tejero et al. (2008); Ugulu et al. (2009); Yöney et al. (2010); Adamidou, (2012); Akaydin et al. (2013); Sargin et al. (2013); Sargin et al. (2015a); Axiotis et al. (2018); Petrakou et al. (2020)
^b^ *Rubus canescens* DC.	Rosaceae			+	G3 (3), G5 (1), G6 (3), G22 (5), G33 (3), G34 (2), G37 (1)	Vokou et al. (1993); Yeşilada et al. (1999); Tuzlaci and Tolon, (2000); Tuzlacı and Aymaz, (2001); Kültür, (2007); Tuzlaci and Alparslan, (2007); Kargioǧlu et al. (2008); Ugulu et al. (2009); Bulut and Tuzlacı, (2015); Akbulut et al. (2019); Karaköse et al. (2019); Sargin and Büyükcengiz, (2019)
^b^ *Rubus fruticosus* L. ex Dierb. (synonym of *Rubus vulgaris* Weihe & Nees)	Rosaceae				G17 (1)	Malamas and Marselos, (1992)
^b^ *Rubus sanctus* Schreb.	Rosaceae			+	G3 (2), G6 (5), G9 (3), G17 (3), G22 (8), G33 (10), G34 (1)	Axiotis et al. (2018); Çakilcioǧlu and Türkoğlu, (2009); Güzel et al. (2015); Yeşilada et al. (1999); Ezer and Mumcu Arisan, (2006); Uzun and Kaya, (2016); Tuzlacı and Bulut, (2007); Tuzlacı and Ii̇, (2011); Bulut and Tuzlacı, (2015); Tuzlacı and Erol, (1999); Sargin et al. (2015a); Ecevit Genç and Özhatay, (2006); Bulut, (2016); Tuzlacı and Sadıkoğlu, (2007); Yeşilyurt et al. (2017); Bulut and Tuzlaci, (2013); Tuzlaci and Tolon, (2000); Honda et al. (1996); Bulut et al. (2017a); Cakilcioglu et al. (2007)
^a^ *Rubus* sp.	Rosaceae			+	G5 (1), G6 (2), G22 (2)	Adamidou, (2012); Kalankan et al. (2015); Karcı et al. (2017)
^b^ *Rumex crispus* L.	Polygonaceae			+	G5 (4), G8 (1), G9 (3), G17 (1), G22 (6), G27 (1), G33 (4), G36 (2)	Axiotis et al. (2018); Altundag and Ozturk, (2011); Karakaya et al. (2019); Karakaya et al. (2020); Petrakou et al. (2020); Everest and Ozturk, (2005); Yeşilada et al. (1995); Kültür, (2007); Günbatan et al. (2016); Tuzlaci et al. (2010); Korkmaz and Si̇, (2015); Ecevit Genç and Özhatay, (2006); Özgen et al. (2012); Ertuǧ, (2000); Özgökçe and Özçelik, (2004)
^b^ *Rumex kerneri* Borbás (synonym of *Rumex cristatus* subsp. *kerneri* (Borbás) Akeroyd & D.A.Webb)	Polygonaceae				G2 (1), G34 (1)	Brussell, (2004)
^c^ *Ruscus aculeatus* L.	Asparagaceae			+	G8 (1), G17 (1), G22 (1), G33 (1)	Tuzlacı and Aymaz, (2001); Hanlidou et al. (2004)
^a^ *Ruta graveolens* L.	Rutaceae			+	G16 (1), G27 (1)	Hanlidou et al. (2004); Kültür, (2007)
^a^ *Salix alba* L.	Salicaceae			+	G4 (2), G5 (2), G9 (1), G17 (1), G19 (1), G20 (1), G32 (2), G33 (1)	Yeşilada et al. (1995); Ezer and Mumcu Arisan, (2006); Kültür, (2007); Sargin et al. (2013); Han and Bulut, (2015); Polat et al. (2015); Axiotis et al. (2018); Karakaya et al. (2020); Petrakou et al. (2020)
^b^ *Salvia fruticosa* Mill.	Lamiaceae		+	+	G2 (1), G4 (5), G6 (2), G18 (1), G32 (1), G33 (1), G35 (1), G37 (1)	Hanlidou et al. (2004); Pieroni et al. (2005b); Everest and Ozturk, (2005); González-Tejero et al. (2008); Karousou and Deirmentzoglou, (2011); Gürdal and Kültür, (2013); Axiotis et al. (2018)
^a^ *Salvia officinalis* L.	Lamiaceae				G33 (1)	Tsioutsiou et al. (2017)
^a^ *Salvia* sp.	Lamiaceae				G2 (1), G4 (1), G6 (1), G33 (1)	Petrakou et al. (2020)
^a^ *Sambucus ebulus* L.	Adoxaceae	+		+	G3 (1), G4 (3), G5 (1), G8 (2), G9 (4), G10 (4), G16 (4), G17 (4), G20 (1), G22 (9), G23 (1), G27 (1), G33 (11), G36 (2)	Sezik et al. (1992); Fujita et al. (1995); Yeşilada et al. (1995); Honda et al. (1996); Yeşilada et al. (1999); Tuzlaci and Tolon, (2000); Tuzlacı and Aymaz, (2001); Brussell, (2004); Ecevit Genç and Özhatay, (2006); Kültür, (2007); Tuzlaci and Alparslan, (2007); Demirci and Özhatay, (2012); Pieroni et al. (2014b); Pieroni et al. (2014c); Güneş et al. (2017); Karcı et al. (2017); Pieroni and Sõukand, (2017); Gürbüz et al. (2019)
^a^ *Sambucus nigra* L.	Adoxaceae	+	+	+	G4 (8), G5 (2), G6 (1), G9 (3), G10 (1), G17 (2), G20 (2), G22 (5), G28 (1), G32 (3), G33 (8), G36 (1)	Malamas and Marselos, (1992); Sezik et al. (1997); Hanlidou et al. (2004); Ecevit Genç and Özhatay, (2006); Pieroni et al. (2006); Kültür, (2007); Yöney et al. (2010); Altundag and Ozturk, (2011); Karousou and Deirmentzoglou, (2011); Adamidou, (2012); Lardos and Heinrich, (2013); Pieroni et al. (2014a); Pieroni et al. (2014b); Güler et al. (2015a); Ahmet Sargin, (2015); Sargin et al. (2015a); Pieroni and Sõukand, (2017); Yeşilyurt et al. (2017); Sargin and Büyükcengiz, (2019); Tsioutsiou et al. (2019); Petrakou et al. (2020)
*Santalum album* L.	Santalaceae				G4 (1)	Hanlidou et al. (2004)
^a^ *Saponaria officinalis* L.	Caryophyllaceae				G2 (1), G3 (1), G4 (1), G17 (2), G20 (1), G23 (1)	Brussell, (2004); Hanlidou et al. (2004); Tsioutsiou et al. (2019)
^a^ *Satureja thymbra* L.	Lamiaceae				G4 (1), G21 (1), G33 (1)	Hanlidou et al. (2004); Axiotis et al. (2018); Petrakou et al. (2020)
*Scandix pecten-veneris* L.	Apiaceae				G4 (1)	Axiotis et al. (2018)
^b^ *Scrophularia canina* L.	Scrophulariaceae				G20 (1), G31 (1), G33 (1)	Brussell, (2004); Axiotis et al. (2018)
^a^ *Scrophularia* sp.	Scrophulariaceae				G8 (1), G17 (1), G27 (1)	Petrakou et al. (2020)
^a^ *Sideritis* sp.	Lamiaceae				G37 (1)	Petrakou et al. (2020)
^a^ *Sinapis alba* L.	Brassicaceae				G20 (1), G33(1)	Hanlidou et al. (2004); Petrakou et al. (2020)
*Smilax officinalis* Kunth	Smilacaceae				G27 (1)	Petrakou et al. (2020)
^b^ *Solanum dulcamara* L.	Solanaceae			+	G23 (1), G33 (2)	Brussell, (2004); Tuzlacı and Doğan, (2010); Altundag and Ozturk, (2011)
*Sorbus domestica* L.	Rosaceae			+	G3 (1), G33 (2)	Sezik et al. (1997); Brussell, (2004); Altundag and Ozturk, (2011)
*Spiraea japonica* L.f.	Rosaceae				G12 (1)	Hanlidou et al. (2004)
^b^ *Stellaria media* (L.) Vill.	Caryophyllaceae				G8 (2), G17 (1), G27 (2), G33 (1)	Axiotis et al. (2018); Petrakou et al. (2020)
*Stereospermum suaveolens* DC. [synonym of *Stereospermum chelonoides* (L.f.) DC.]	Bignoniaceae				G4 (1)	Hanlidou et al. (2004)
^b^ *Symphytum bulbosum* K.F.Schimp.	Boraginaceae				G28 (1)	Brussell, (2004)
^b^ *Symphytum ottomanum* Friv.	Boraginaceae				G20 (1), G22 (1), G33 (1)	Hanlidou et al. (2004); Petrakou et al. (2020)
^a^ *Syringa vulgaris* L.	Oleaceae				G21 (1)	Hanlidou et al. (2004)
*Tamus communis* L. (synonym of *Dioscorea communis* (L.) Caddick & Wilkin)	Dioscoreaceae			+	G10 (1), G17 (1), G22 (1), G33 (3)	Tuzlacı and Aymaz, (2001); Bulut and Tuzlaci, (2013); Bulut and Tuzlacı, (2015); Axiotis et al. (2018)
*Tanacetum vulgare* L.	Asteraceae				G36 (1)	Brussell, (2004)
^b^ *Taraxacum hellenicum* Dahlst.	Asteraceae				G34 (1)	Axiotis et al. (2018)
^a^ *Taraxacum* sp.	Asteraceae				G1 (2), G9 (1), G13 (1), G17 (2), G20 (1)	Hanlidou et al. (2004); Papageorgiou et al. (2020); Petrakou et al. (2020)
^a^ *Teucrium chamaedrys* L.	Lamiaceae			+	G6 (3), G8 (1), G17 (1), G22 (12), G33 (4), G36 (1)	Yeşilada et al. (1993), Fujita et al. (1995), Brussell (2004), Ecevit Genç and Özhatay (2006), Sarper et al. (2009), Cakilcioglu and Turkoglu (2010), Ünsal et al. (2010), Demirci and Özhatay (2012), Uysal et al. (2012), Özgen et al. (2012), Ahmet Sargin (2015), Ari et al. (2015), Bulut and Tuzlacı (2015), Özdemir and Alpınar (2015), Paksoy et al. (2016), Bulut et al. (2017a), Güneş et al. (2017)
^a^ *Teucrium polium* L.	Lamiaceae			+	G3 (2), G5 (1), G9 (2), G17 (5), G22 (15), G28 (1), G33 (1), G36 (1)	Sezik et al. (1992), Honda et al. (1996), Sezik et al. (1997), Yeşilada et al. (1999), Hanlidou et al. (2004), Everest and Ozturk (2005), Tuzlaci et al. (2010), Ünsal et al. (2010), Altundag and Ozturk (2011), Adamidou (2012), Bulut and Tuzlaci (2013), Hayta et al. (2014), Ari et al. (2015), Bulut and Tuzlacı (2015), Han and Bulut (2015), Kalankan et al. (2015), Günbatan et al. (2016), Karakaya et al. (2019)
^b^ *Thymbra capitata* (L.) Cav. (= *Thymus capitatus* (L.) Hoffmanns. & Link)	Lamiaceae		+	+	G4 (3), G6 (1), G8 (1), G32 (1), G33 (2)	González-Tejero et al. (2008), Yöney et al. (2010), Sargin and Büyükcengiz (2019), Papageorgiou et al. (2020), Petrakou et al. (2020)
^a^ *Thymus* sp.	Lamiaceae			+	G2 (1), G4 (2)	Hanlidou et al. (2004), Everest and Ozturk (2005), Akgül et al. (2016), Karcı et al. (2017)
*Tilia* sp.	Malvaceae				G2 (1), G4 (1), G13 (1), G33 (1), G37 (1)	Hanlidou et al. (2004), Adamidou (2012), Petrakou et al. (2020)
^a^ *Tribulus terrestris* L.	Zygophyllaceae			+	G4 (2), G5 (1), G6 (1), G17 (3), G20 (2), G22 (4), G36 (1)	Axiotis et al. (2018), Tetik et al. (2013), Ari et al., (2015), Tuzlacı and Ii̇ (2011), Sargin et al. (2013), Sargin et al. (2015a), Ahmet Sargin (2015), Tuzlacı and Sadıkoğlu (2007), Bulut and Tuzlaci (2013)
^a^ *Trifolium pratense* L.	Leguminosae			+	G17 (2), G27 (2), G33 (2)	Sezik et al. (1997), Hanlidou et al. (2004), Altundag and Ozturk (2011), Petrakou et al. (2020)
^a^ *Trigonella foenum-graecum* L.	Leguminosae			+	G1 (1), G8 (1), G9 (1), G33 (1)	Ugulu et al. (2009), Petrakou et al. (2020)
^a^ *Tussilago farfara* L.	Asteraceae	+	+	+	G5 (4), G8 (1), G9 (2), G20 (1), G33 (8)	Yeşilada et al. (1995), Yeşilada et al. (1999), Tuzlacı and Aymaz (2001), Hanlidou et al. (2004), Uzun et al. (2004), Pieroni et al. (2005a), Pieroni et al. (2005b), Ugulu et al. (2009), Karousou and Deirmentzoglou (2011), Tetik et al. (2013), Bulut and Tuzlacı (2015), Kalankan et al. (2015), Özdemir and Alpınar (2015)
*Ulmus minor* Mill.	Ulmaceae	+		+	G1 (1), G2 (1), G5 (1), G9 (1), G30 (1), G33 (4), G36 (1)	Fujita et al. (1995), Yeşilada et al. (1999), Ecevit Genç and Özhatay (2006), Altundag and Ozturk (2011), Pieroni and Sõukand (2017), Petrakou et al. (2020)
*Urospermum picroides* (L.) Scop. ex F.W.Schmidt	Asteraceae				G4 (1)	Axiotis et al. (2018)
^a^ *Urtica dioica* L.	Urticaceae	+		+	G1 (1), G2 (16), G3 (3), G4 (3), G5 (5), G8 (5), G9 (3), G10 (5), G14 (2), G16 (1), G17 (13), G22 (26), G27 (1), G31 (1), G32 (2), G33 (8), G34 (1), G36 (5)	Nadiroğlu et al. (2019); Vokou et al. (1993), Fujita et al. (1995), Tuzlacı and Erol (1999), Yeşilada et al. (1999), Tuzlaci and Tolon (2000), Sezik et al. (2001), Tuzlacı and Aymaz (2001), Uzun et al. (2004), Pieroni et al. (2005a), Pieroni et al. (2005b), Ecevit Genç and Özhatay (2006), Ezer and Mumcu Arisan (2006), Kültür (2007), Tuzlaci and Alparslan (2007), Tuzlacı and Sadıkoğlu (2007), Yildirim et al. (2008), Ugulu et al. (2009), Çakilcioǧlu and Türkoğlu (2009), Tuzlaci et al. (2010), Altundag and Ozturk (2011), Polat and Satıl (2012), Uysal et al. (2012), Akaydin et al. (2013), Tetik et al. (2013), Pieroni et al. (2014a), Sargin et al. (2015a), Güler et al. (2015b), Bulut and Tuzlacı (2015), Güzel et al. (2015), Polat et al. (2015), Akgül et al. (2016), Bulut (2016), Günbatan et al. (2016), Uzun and Kaya (2016), Karcı et al. (2017), Pieroni (2017), Tsioutsiou et al. (2017), Yeşilyurt et al. (2017), Axiotis et al. (2018), Gürbüz et al. (2019), Güler et al. (2020)
^a^ *Urtica* sp.	Urticaceae		+	+	G2 (4), G3 (3), G4 (1), G14 (1), G17 (2), G22 (3), G33 (1), G37 (1)	Hanlidou et al. (2004), Everest and Ozturk (2005), Karousou and Deirmentzoglou (2011), Adamidou (2012), Papageorgiou et al. (2020), Petrakou et al. (2020)
^a^ *Urtica urens* L.	Urticaceae		+	+	G2 (1), G5 (1), G9 (2), G17 (3), G20 (1), G22 (3), G28 (1), G32 (2), G33 (3)	Fujita et al. (1995); Tuzlacı and Erol (1999), Tuzlacı and Sadıkoğlu (2007), González-Tejero et al. (2008), Tuzlaci et al. (2010); Akaydin et al. (2013), Charalampidou (2014), Ahmet Sargin (2015), Ari et al. (2015), Han and Bulut (2015), Kalankan et al. (2015), Tsioutsiou et al. (2017)
^c^ *Valeriana officinalis* L.	Caprifoliaceae			+	G33 (2)	Karaman and Kocabas (2001), Hanlidou et al. (2004)
^b^ *Verbascum mucronatum* Lam.	Scrophulariaceae				G4 (1), G8 (1)	Axiotis et al. (2018)
^a^ *Verbascum* sp.	Scrophulariaceae	+		+	G3 (1), G4 (1), G5 (1), G17 (1), G20 (1), G22 (3), G32 (1), G33 (2), G36 (1)	Hanlidou et al. (2004), Pieroni et al. (2005a), Akaydin et al. (2013), Sargin et al. (2013), Tetik et al. (2013), Ahmet Sargin (2015), Ari et al. (2015), Pieroni (2017)
*Vinca major* L.	Apocynaceae				G1 (1), G3 (1), G4 (1), G16 (1), G17 (1)	Axiotis et al. (2018)
^a^ *Vinca minor* L.	Apocynaceae				G3 (1)	Hanlidou et al. (2004)
^b^ *Viola macedonica* Boiss & Heldr.	Violaceae				G17 (1)	Petrakou et al. (2020)
^a^ *Vitex agnus-castus* L.	Lamiaceae			+	G4 (1), G5 (1), G9 (1), G10 (1), G15 (1), G16 (2), G17 (2), G20 (1), G22 (2), G28 (2), G33 (2)	Yeşilada et al. (1995), Brussell (2004), Tuzlacı and Sadıkoğlu (2007), Ugurlu and Secmen (2008), Ugulu et al. (2009) Kargıoğlu et al. (2010), Adamidou (2012), Polat and Satıl (2012), Bulut and Tuzlaci (2013), Gürdal and Kültür (2013), Bulut and Tuzlacı (2015), Güzel et al. (2015)
^a^ *Vitis vinifera* L. (= *Vitis sylvestris* C.C.Gmel.)	Vitaceae	+	+	+	G1 (1), G2 (4), G4 (2), G5 (1), G6 (1), G9 (6), G10 (2), G14 (1), G16 (1), G22 (4), G25 (1), G29 (1), G32 (2), G33 (8)	Yeşilada et al. (1995); Tuzlacı and Erol (1999), Karaman and Kocabas (2001), Sezik et al. (2001), Pieroni et al. (2005b); Kültür (2007), Çakilcioǧlu and Türkoğlu (2009), Yöney et al. (2010), Kizilarslan and Özhatay (2012), Lardos and Heinrich (2013), Sargin et al. (2013), Sargin et al. (2015a), Güzel et al. (2015), Günbatan et al. (2016), Güneş et al. (2017), Pieroni and Sõukand (2017), Axiotis et al. (2018), Gürbüz et al. (2019)
*Zingiber officinale* Roscoe	Zingiberaceae			+	G2 (1), G4 (1), G20 (1)	Hanlidou et al. (2004), Güzel et al. (2015), Petrakou et al. (2020)
*Ziziphora taurica* M.Bieb.	Lamiaceae			+	G33 (2)	Cakilcioglu et al. (2007); Axiotis et al. (2018)

±Ailment category for which the specific plant is used, as well as the number of reported uses in all four countries, in parenthesis.

a Common taxa reported in the ethnobotanical studies conducted in Greece and in Dioscorides’ *De Materia Medica*.

b Common taxa reported in the ethnobotanical studies conducted in Greece and in Dioscorides’ *De Materia Medica*-same genus, not same species.

c Common taxa reported in the ethnobotanical studies conducted in Greece and in Dioscorides’ *De Materia Medica*-in *De Materia Medica* not used against skin disorders.

The percentage of common taxa reported between the ethnobotanical studies conducted in Greece and Albania is 14.4% (31 taxa), Greece and Cyprus is 22.8% (49 taxa), Greece and Turkey is 63.3% (136 taxa), while between Greece and those mentioned in Dioscorides’ *De Materia Medica* is 48.8% (105 taxa). The percentage of common taxa reported between the ethnobotanical studies conducted in Greece and those conducted in Albania and Cyprus is low, even though they are countries with high historical and cultural connections, as aforementioned. This can be justified considering that since not many ethnobotanical studies have been carried out in Albania (7 studies) and Cyprus (5 studies), many plants have not yet been recorded, even though they may be used for the treatment of skin diseases nowadays. This conclusion can be strengthened by the fact that only 29 and 40 different families including 60 and 82 different taxa respectively have been reported in these two countries up to now.

On the other hand, even though the number of ethnobotanical studies conducted in Turkey (103 studies with 859 different taxa) is vastly higher than those conducted in Greece (13 studies with 215 different taxa), the percentage of common taxa reported is high. This could be due to geomorphological factors, floristic similarities, as well as historical and cultural reasons. Turkey is part of the continent of Asia and Europe, while Greece represents the tip of a peninsula appertaining to the continent of Europe. Greece, in spite of its small territory, has the richest flora in Europe, in terms of plant biodiversity per area unit and one of the richest worldwide. The wide geological history, the presence of different rock substrates (limestones, schists, and granite serpentine) and the complicated topography represent some of the factors that contribute to the floristic variety and diversity (Strid, 1986). The Greek flora consists of at least 6.700 species and subspecies and over 22% are endemic (Dimopoulos et al., 2016). Turkey, on the other hand, extends through a vast geographical area including coastal landmarks (Mediterranean and Black sea), dessert plains, lakes and highlands with mountain steppes (Kuzucuoğlu et al., 2019). A considerable number of the Greek mountain plants are also found in Turkey, while taxa restricted to the Balkan Peninsula and Anatolia constitute between 12 and 22% of the narrowly distributed taxa or between 4 and 9% of the total mountain flora of Greece. The Anatolian element is mostly represented in the North East and in Crete (22 and 21% of the “narrows” respectively) and is significantly smaller in the Pindhos and North Central (12%–14%). The percentage of “Turkish” species in the Greek mountain flora is thus roughly three times as high as the percentage of “Greek” species in the Turkish mountain flora. The migratory pressure from east to west is much greater than that from west to east (Strid, 1986). Moreover, inhabitants of the European part, as well as those of the Mediterranean coastline of Turkey have been in constant contact with people from the Balkans through trade and in relation to many historical facts. As such, there has been a reciprocal influence throughout the ages concerning traditional medicine and other cultural and social traditions. Inhabitants of East- and Southeastern Anatolia on the other hand were mostly influenced, both commercially and culturally, by Asian populations due to the constant flow of trade along the Silk Roads (MA, 2014). Since ethnobotanical studies included Turkish populations deriving from the whole Turkish domain, both European and Asian, it is somewhat expected that traditional medicine of Turkey is comprised by a blend of all these elements and cultures. Despite the different territorial size between Turkey and Greece, the floristic, historical, and cultural correlation lead to an important common number of species present in the ethnobotanical studies conducted in both countries.

Concerning the comparison between taxa reported in the ethnobotanical studies conducted in Greece and the suggested plants regarding the treatment of skin ailments reported in Dioscorides’ *De Materia Medica*, the percentage of common ones is 50%. Out of 215 different taxa reported in Greek ethnobotanical filed studies, 105 taxa were common, whereas 105 were not mentioned in *De Materia Medica*, yet 36 are only mentioned as genera. Moreover, 5 species occurring in the Greek studies are mentioned in the ancient manuscript but are not reported for skin related ailments. Furthermore, Greek traditional medicine, as well as other social and cultural aspects have been influenced by many different peoples, not only through commercial trade, but also due to occupation. From Byzantium to Francs and the Ottoman Empire, there has been a blending of all these different traditions and cultures through centuries. Additionally, Dioscorides refers to treatments against many skin ailments also present today, creating a strong bond between the past and the present. The comparison between the information obtained through the bibliographical analysis of the ethnobotanical research and Dioscorides’ manuscript, led to the conclusion that many of the remedies recommended against skin diseases in *De Materia Medica*, are also used as herbal therapies in the four countries for the treatment of the same skin conditions (Dioscorides et al., 2000) (Table 3). However, the data of this comparison will change over time, since few ethnobotanical studies have been carried out in the four countries on the topic up to now. The limited number of surveys should raise concern because many Greek populations, especially in remote areas, still possess this vital knowledge. Although their experience has not been recorded, it is transmitted through generations orally.

## Conclusion

In the present review, an extensive literature search was performed concerning published ethnobotanical field studies conducted in Albania, Cyprus, Greece, and Turkey up until May 2020, collecting data from 128 published articles concerning skin related ailments. This documentation can significantly contribute to the preservation of the ethnobotanical knowledge of the study area, since it is the first time that such a data collection was catalogued and statistically elaborated. Our findings suggest that traditional medicine plays an important role in the culture of Albanians, Cypriots, Greeks, and Turks and that the four populations, related historically and culturally, are demonstrated to have a common background on the use of medicinal plants against various skin diseases. The analysis showed that there is a substantial necessity to carry out more ethnobotanical field studies in this area but also in other countries of the Balkan Peninsula and the Mediterranean Sea to reveal more medical practices and treatment remedies not yet encountered. Moreover, the extended study of Dioscorides’ *De Materia Medica* verifies the consensus that ancient herbals and their manuscripts have influenced and guided the development of Mediterranean and European traditional herbal medicine. This is confirmed by the number of species commonly mentioned and used in both ethnopharmacological surveys and Dioscorides’ plant descriptions. As a result, this can give rise to delving into other important herbal manuscripts enabling them as sources of evidence deriving from the past, and to evaluate the traditional medical practices described, not only against skin disorders, but also for the treatment of other ailments.
